# Dose-response relationship of in vivo ambulatory load and mechanosensitive cartilage biomarkers—The role of age, tissue health and inflammation: A study protocol

**DOI:** 10.1371/journal.pone.0272694

**Published:** 2022-08-19

**Authors:** Simon Herger, Werner Vach, Corina Nüesch, Anna-Maria Liphardt, Christian Egloff, Annegret Mündermann

**Affiliations:** 1 Department of Orthopaedics and Traumatology, University Hospital Basel, Basel, Switzerland; 2 Department of Spine Surgery, University Hospital Basel, Basel, Switzerland; 3 Department of Biomedical Engineering, University of Basel, Basel, Switzerland; 4 Department of Clinical Research, University of Basel, Basel, Switzerland; 5 Basel Academy for Quality and Research in Medicine, Basel, Switzerland; 6 Department of Internal Medicine 3 –Rheumatology and Immunology, Friedrich-Alexander-Universität Erlangen-Nürnberg (FAU), Universitätsklinikum Erlangen, Erlangen, Germany; 7 Deutsches Zentrum für Immuntherapie (DZI), Friedrich-Alexander-Universität Erlangen-Nürnberg (FAU), Universitätsklinikum Erlangen, Erlangen, Germany; GERMANY

## Abstract

**Objective:**

To describe a study protocol for investigating the in vivo dose-response relationship between ambulatory load magnitude and mechanosensitive blood markers of articular cartilage, the influence of age, cartilage tissue health and presence of inflammation on this relationship, and its ability to predict changes in articular cartilage quality and morphology within 2 years.

**Design:**

Prospective experimental multimodal (clinical, biomechanical, biological) data collection under walking stress and three different load conditions varied in a randomized crossover design.

**Experimental protocol:**

At baseline, equal numbers of healthy and anterior cruciate ligament injured participants aged 20–30 or 40–60 years will be assessed clinically and complete questionnaires regarding their knee health. Biomechanical parameters (joint kinetics, joint kinematics, and surface electromyography) will be recorded while performing different tasks including overground and treadmill walking, single leg balance and hopping tasks. Magnetic resonance images (MRI) of both of knees will be obtained. On separate stress test days, participants will perform a 30-minute walking stress with either reduced (80% body weight (BW)), normal (100%BW) or increased (120%BW) load. Serum blood samples will be taken immediately before, immediately after, 30, 120 and 210 minutes after the walking stress. Concentration of articular cartilage blood biomarkers will be assessed using enzyme linked immunosorbent assays. At 24-month follow-up, participants will be again assessed clinically, undergo an MRI, complete questionnaires, and have a blood sample taken.

**Conclusion:**

The study design provides a standardized set up that allows to better understand the influence of ambulatory load on articular cartilage biomarkers and thereby extend current knowledge on in vivo cartilage metabolism and mechanosensitivity. Further, this study will help to elucidate the prognostic value of the load-induced cartilage biomarker response for early articular cartilage degeneration.

**Trial registration:**

The protocol was approved by the regional ethics committee and has been registered at clinicaltrials.gov (NCT04128566).

## Introduction

In most Western countries including the US, more than 9% of the population are estimated to be diagnosed with symptomatic knee osteoarthritis (OA) by the age of 60 years [1]. Several factors including age, female sex, obesity [1,2] and previous knee injury [2–4] such as anterior cruciate ligament (ACL) rupture [5] increase the risk for knee OA. The pathogenic progression from a healthy to an osteoarthritic joint happens before symptoms of OA, such as joint pain [2,6], joint swelling, crepitus, movement limitations [7] and different forms of inflammations [8], are recognized. OA is a complex whole organ disease with pathological changes in cartilage, subchondral bone and synovium [9,10]. In advanced OA stages, structural changes such as joint osteophytes (Kellgren-Lawrence (KL) grade 2 or higher), and joint space narrowing (KL grade 3 or higher) can be confirmed radiologically [11] or by magnetic resonance imaging (MRI) [12]. MRI has the advantage that especially cartilage [13] and other organ tissues are visualized in 3D [12] and enables semi-quantitative whole organ assessments such as WORMS [14] or MOAKS [15]. Modern MRI sequences such as double-echo steady-state (DESS) sequences may be promising for detecting even early-stages of OA [16]. However, acquiring MRI is time and cost intensive [12] and only sensitive to cartilage changes in the range of months or years, and hence alternative strategies for detecting early-stages of OA are desirable.

Articular cartilage is avascular and aneural [17], and transport of nutrients and waste products in and out of cartilage occurs through diffusion [18]. Under normal physiological conditions, chondrocytes synthesize and maintain crucial extracellular matrix (ECM) components that confer the functional properties of cartilage [17]. Main components of the ECM are water, collagens (90% to 95% Type II), sulphated proteoglycans and non-collagenous proteins [17,19–21]. Under pathological conditions such as OA, chondrocytes exhibit an imbalance of anabolic and catabolic activities that are characterized by degenerative changes in the cartilage matrix and other joint tissues, including the subchondral bone and synovium [9,22,23]. Although the individual influence of molecular mechanisms that trigger the pathological changes in the initiation of OA are largely unknown, the ability of chondrocytes to respond to load is believed to play a critical role for maintaining healthy tissue and in the initiation of OA further emphasizing the importance of ambulatory load in OA pathophysiology [24–26].

Systemic blood biomarkers are frequently investigated as surrogates for the in vivo articular cartilage metabolism [27,28]. Candidates for mechanosensitive blood markers of articular cartilage include cytokines, enzymes involved in ECM metabolism, non-collagenous proteins and products of collagen synthesis or degradation [29–31]. In a pilot study, we established an experimental framework to modulate ambulatory load [32] and showed that the increase in concentration of serum cartilage oligomeric matrix protein (sCOMP) [33] and serum matrix metalloproteinase (sMMP)-3 [34] depend on the magnitude of the applied load. Further, serum interleukin (sIL)-6 increased after the walking stress independent of the ambulatory load magnitude [34].

Despite these promising results, overall, the dose-response relationship between ambulatory load magnitude and mechanosensitive blood markers of articular cartilage is poorly understood raising the following questions:

1. Is there a biological variation in the dose-response relationship between ambulatory load and mechanosensitive blood markers of articular cartilage and to which degree can it be explained by age, articular cartilage tissue health (termed: tissue health) or the presence of inflammation?
2. Does the individual dose-response relationship between ambulatory load and mechanosensitive blood markers of articular cartilage predict future cartilage degeneration in persons at risk for developing early OA?

Answering these questions will provide information that helps to sort out the processes underlying the in vivo mechanosensitivity of articular cartilage and its potential role in the initiation of knee OA. Here, we describe a study protocol for providing first evidence of the role of age, tissue health and presence of systemic inflammation on the dose-response relationship between in vivo ambulatory load and concentration of mechanosensitive blood markers of articular cartilage and its relevance for predicting cartilage degeneration. Because of the exploratory character of this study, we will include subjects with a variability in age and in knee tissue health.

### Specific Aim 1

To investigate the in vivo dose-response relationship of weight bearing and mechanosensitive blood markers of articular cartilage using controlled weight bearing during a walking stress test and age, tissue health and the presence of inflammation as experimental paradigms.

**Hypothesis 1:** The slope of the relationship between ambulatory load and mechanosensitive blood markers of articular cartilage will be smaller in:
  1.1 older participants than in younger participants.
  1.2 participants with lower cartilage quality than those with higher cartilage quality.
  1.3 participants with signs of inflammation than those with no signs of inflammation.

We expect that with older age or after injury–both major risk factors for the development of OA–the response of articular cartilage to ambulatory load will change.

**Specific Aim 2:** To investigate the prognostic ability of the individual in vivo dose-response relationship of ambulatory load and mechanosensitive blood markers of articular cartilage for articular cartilage degeneration.

**Hypothesis 2:** The slope of the relationship between ambulatory load and mechanosensitive blood markers of articular cartilage will negatively correlate with:
  2.1 subsequent change in articular cartilage T2 relaxation time within 2 years.
  2.2 subsequent articular cartilage thinning within 2 years.
  2.3 the change in Knee Injury and Osteoarthritis Outcome Score (KOOS) and modified Knee Society Score (KSS) within 2 years.

We expect that altered mechanosensitivity will lead to articular cartilage degeneration measured as articular cartilage thinning and decrease in articular cartilage quality and that physical activity level modulates this relationship.

## Materials and methods

Here, we describe the study design, participant characteristics, procedures and methods. In the subsequent Discussion section, we will provide the rationale for specific aspects of our study protocol.

### Study design

**Fig 1:**
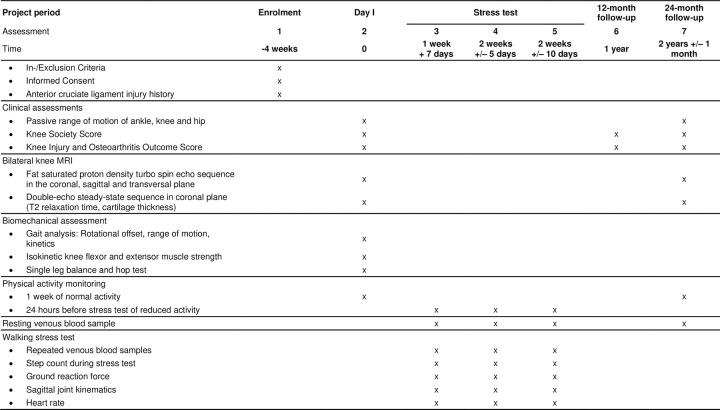
Schedule of enrollment and assessments.

This study comprises prospective experimental multimodal (clinical, biomechanical, biological; Fig 1) data collection during walking stress under three different load conditions with either reduced (80% body weight (BW)), normal (100%BW) or increased (120%BW) load. Each participant is exposed to all three conditions in a cross over design. Within each of the four participant groups, participants are block randomized to the 6 possible orders of the loading condition with a block size of 6.

### Participants

**Fig 2:**
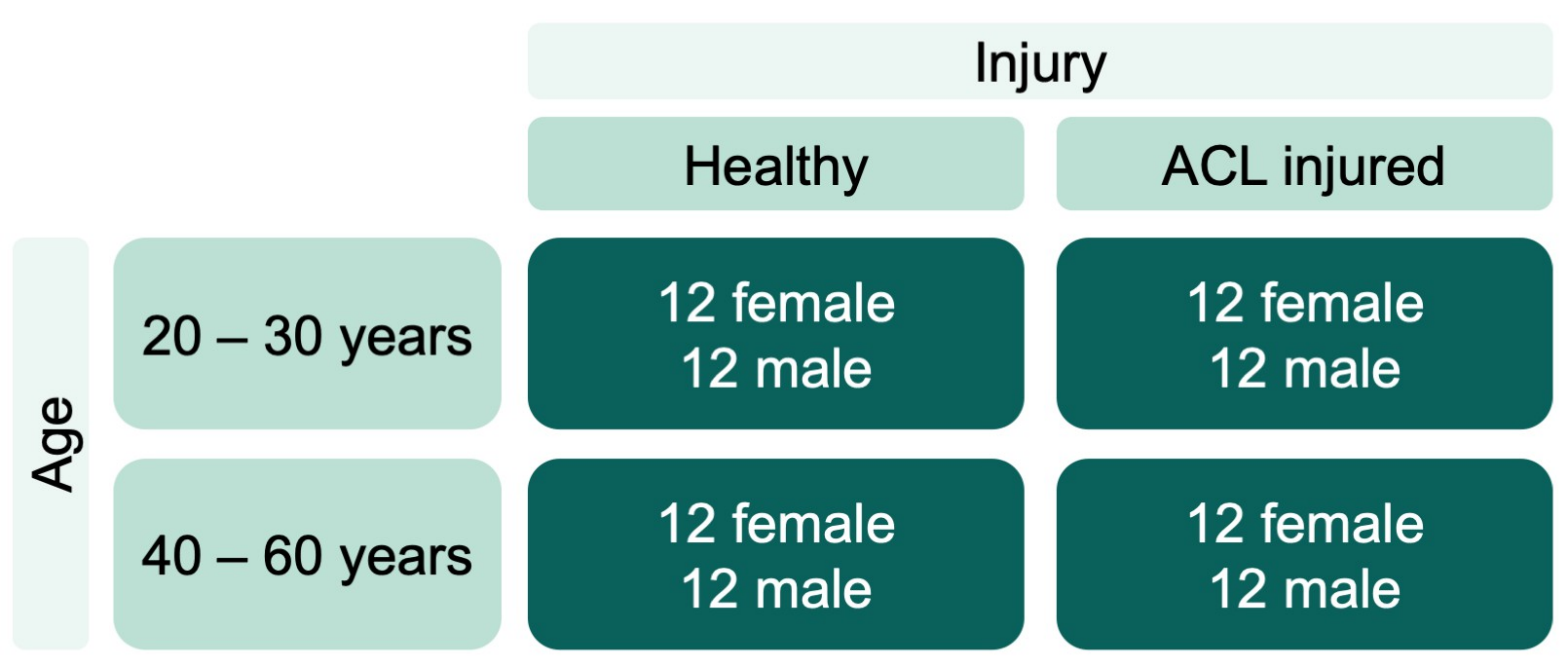
Planned participant characteristics. Injury subcategories healthy and ACL injured and the age subcategories 20 to 30 and 40 to 60 years. ACL–Anterior cruciate ligament.

We will examine a cohort of 96 participants (Fig 2). Experiments for all sub aims will involve the same participants (n = 24 per group; 12 female, 12 male):

- Group 1: healthy participants aged between 20 and 30 years
- Group 2: participants with previous ACL injury aged between 20 and 30 years
- Group 3: healthy participants aged between 40 and 60 years
- Group 4: participants with previous ACL injury aged between 40 and 60 years

A minimum age of 20 years and maximum age of 60 years ensures skeletal maturity and includes participants with an increased risk of early knee OA (above 40 years [35,36]). Healthy and ACL injured participants will be recruited from the community surrounding our orthopedic clinic and by placing a flyer on the institutional website. Moreover, we will review the patient list of our orthopedic clinic for persons with ACL injuries 2 to 10 years prior to the study. Eligible candidates will be contacted by telephone and asked if they want to receive further information on the study.

**Inclusion and exclusion criteria.** Inclusion criteria and exclusion criteria are listed in Table 1.

**Table 1:** Inclusion and exclusion criteria applied in this study.

*Inclusion criteria*	*Exclusion criteria*
• no major medical problems (describe themselves as healthy) • at least 2 hours of physical activity per week *Groups 1 and 3* • no previous known knee injury *Groups 2 and 4* • ACL rupture 2 to 10 years prior to study participation (operative or conservative treatment)	• inability to provide informed consent • age < 20 years (before maturation) or age > 60 years (because of possible advanced sarcopenia and higher likelihood of osteoarthritic changes) • BMI > 35 kg/m2 (because of excessive skin movement that influences the gait analysis) • inability to walk for 30 minutes • contraindications for a knee MRI • active rheumatic disorder • prior neuromuscular impairment (e.g., stroke) • conditions other than knee injury that could cause abnormal patterns of locomotion • prior hip, knee, and ankle prosthesis • osteotomy of the lower extremities • prior spine surgery • pregnancy • persons who have previously completed or withdrawn from this study • patients currently enrolled in another experimental (interventional) protocol *Groups 2 and 4* • bilateral ACL injury • ACL re-rupture within 2 years after treatment • More than one ACL re-rupture • surgically treated medial or lateral collateral ligament rupture • posterior cruciate ligament rupture • total or partial meniscectomy • bone bruise • fractures in tibia plateau or femur condyles

ACL–anterior cruciate ligament; BMI–Body mass index; MRI–Magnetic resonance imaging.

### Ethical considerations

The experimental protocol was approved (16th August 2019) by the regional ethics board (Ethics Committee Northwest Switzerland EKNZ 2019–01315) and registered at clinicaltrials.gov (NCT04128566). Written informed consent will be obtained from all participants prior to participation. In the informed consent process, participants will be asked if they wish to be informed in case of incidental findings and referred to a specialist.

### Procedures

**Fig 3:**
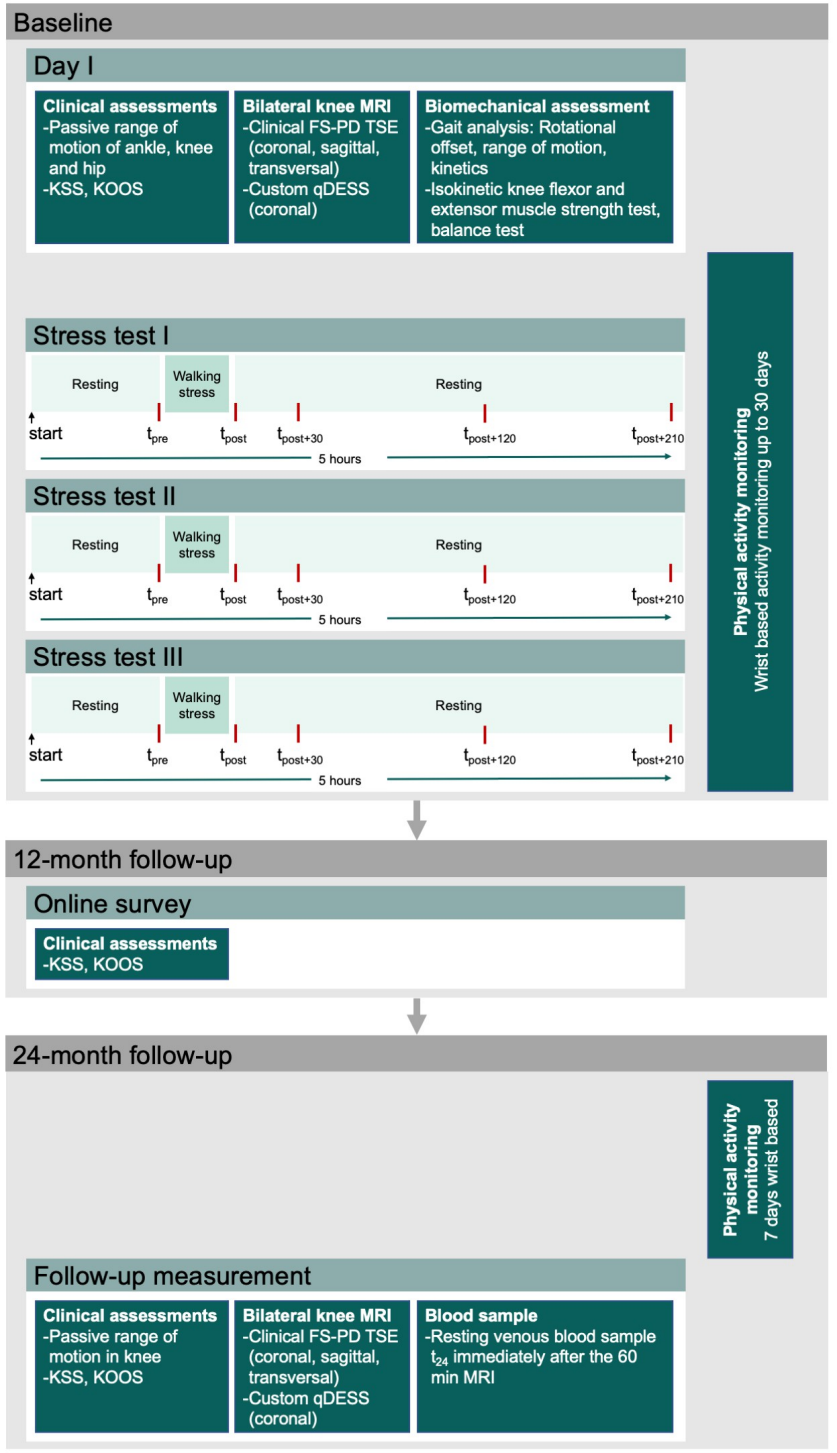
Illustration of the study design. Overview and timeline of the measurements of baseline, 12-month follow-up and 24-month follow-up data collection. KSS–Knee Society Score; KOOS–Knee Injury and Osteoarthritis Outcome Score; MRI–magnetic resonance imaging; FS-PD TSE–fat saturated proton density turbo spin echo; qDESS–quantitative double-echo steady-state.

Data will be collected at baseline, 12-month follow-up and 24-month follow-up (Fig 3).

To address Specific Aim 1, baseline data will be collected in four experimental sessions scheduled on four separate days (day I, stress test days I-III, Fig 3) within 1 month. On day I, participants will complete questionnaires (KOOS, KSS) and be clinically assessed, undergo MRI of both knees and complete gait analysis. Participants will be asked to wear an activity monitor from day I until the end of stress test III. On stress test days I to III, participants will complete a walking stress test. In the walking stress test, participants will walk for 30 minutes on a treadmill (walking stress) with one of the three loading conditions (80%BW, 100%BW and 120%BW). During the walking stress, selected gait parameters and heart rate will be measured. Participants will rest in a seated position 1 hour before and 3.5 hours after the walking stress. Throughout this entire period, blood samples will be obtained at five predefined times (Fig 3). At 12-month follow-up, participants will be asked to complete online questionnaires (KOOS, KSS).

To address Specific Aim 2, at 24-month follow-up (Fig 3) participants will be clinically assessed, undergo MRI of both knees, and a resting blood sample will be taken immediately after the MRI. Participants will be asked to wear an activity monitor for the 7 days prior to the 24-month follow-up.

### Measurements

#### Clinical assessments

The choice of clinical parameters is based on the ICHOM standards [37]. Clinical evaluation will be carried out using the modified KSS and its functional component [38]. The KOOS will be used to assess pain, symptoms, activities of daily living, sport and recreation, and knee related quality of life [39]. Active range of motion of ankle, knee, and hip joint will be measured using a long arm goniometer.

#### Bilateral knee MRI

MRI of both knees will be obtained using a 3T MR scanner (Prisma, Siemens Healthineers, Erlangen, Germany). Each knee will be scanned separately. The knee will be centered in a dedicated knee coil, the popliteal fossa padded allowing a slight knee flexion. Both knees will be imaged using a standard clinical fat saturated proton density turbo spin echo (FS-PD TSE) sequence in the coronal, sagittal and transversal plane. In addition, the articular cartilage of the tibiofemoral joint will be imaged in the coronal plane with an institutional custom quantitative 12-minute 3D DESS sequence [40]. MRI segmentation will be done by a blinded independent service provider (Chondrometrics GmbH medical data processing, Ainring, Germany) using their custom software (WORKS 3.0) [41]. The service provider will not receive any information on the images other than the participant code and random sample numbers to ensure that all image sets of the same participant will be assessed in the same evaluation session. T2 relaxation time and cartilage thickness in loaded regions of tibiofemoral cartilage will be computed and used as surrogates for tissue health. Previous studies reported that T2 relaxation time was higher in females with early OA [42] and in people with mild and severe knee OA [43] than in controls. Tissue health will be assessed by the surrogate measures T2 relaxation time and cartilage thickness where lower T2 relaxation time and greater cartilage thickness correspond to better tissue health.

#### Biomechanical assessment

**Fig 4:**
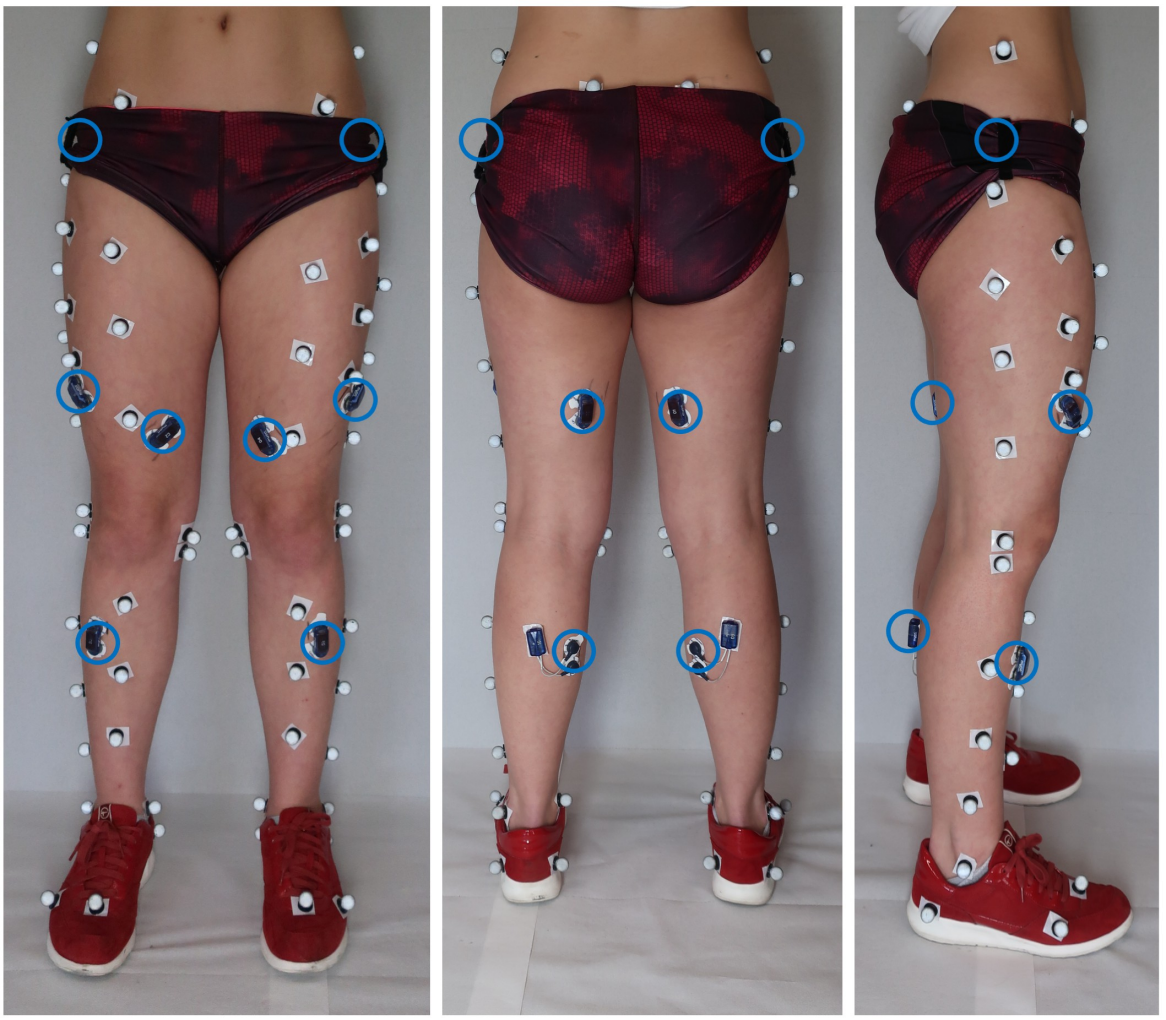
Marker and electromyography electrodes placement during the biomechanical assessment. Surface electromyography electrodes (blue circles; electrodes on gluteus medius under the pants) and marker placement (bright dots) for 3D motion analysis in front (left), back (middle) and side (right) view.

Biomechanical assessments will be carried out on baseline day I after the MRI was taken. Surface electrodes will be placed bilaterally on the gluteus medius, vastus medialis and lateralis, semitendinosus, tibialis anterior, and gastrocnemius medialis muscles following the guidelines of the SENIAM project (Surface ElectroMyoGraphy for the Non-Invasive Assessment of Muscles, Fig 4) [44]. Further, isokinetic muscle strength of knee extensors and flexors will be tested at 60°/s using a dynamometer (Biodex System 4 Pro: Biodex Medical Systems, Shirley, NY, USA) in two series of 4 extension-flexion repetitions. To asses 3D joint kinematics, reflective markers will be placed on predefined anatomical landmarks on the pelvis and lower legs [45] (Fig 4). The participants will then perform a single leg balance testing, an instrumented gait analysis on an overground walkway and on an instrumented treadmill. In all settings, kinematic data will be collected using a 10-camera Vicon system (Vicon, Oxford, UK; frame rate 240 Hz), and electromyographic (EMG) data will be collected using a 12-channel EMG system (myon AG, Schwarzenberg, Switzerland, sampling rate 2400 Hz). For balance and overground walking, ground reaction force (GRF) will be collected using two embedded force plates (Kistler force plate 9260AA6, Kistler AG, Winterthur, Switzerland; sampling rate 2400 Hz). For treadmill walking, vertical ground reaction force (vGRF) will be measured with a plantar pressure plate located under the treadmill belt (h/p/cosmos, Zebris FDM-T, Isny, Germany; 7168 sensors; area, 1.5 * 0.5 m; range, 1–120 N/cm^2^; precision, 1–120 N/cm^2^ ± 5%; sampling rate, 120 Hz), and the participant’s normal walking speed will be recorded for use during the walking stress test. Additionally, single leg hop for distance will be performed while kinematic, kinetic and EMG data is collected. Joint kinematic (and kinetic) trajectories will be computed using pyCGM2 [46,47], normalized to gait cycle and peak values and ranges computed for each setting, leg, and participant. EMG signals will be filtered, full-wave rectified, the linear envelope calculated, and normalized to EMG intensity of the maximum voluntary contractions performed on the dynamometer.

#### Physical activity monitoring

Physical activity will be recorded from baseline day I to stress test day III and 7 days prior the 24-month follow-up visit using an accelerometer worn on the non-dominant wrist (GENEActive Original, Activinsights, Kimbolton, Cambridgeshire, United Kingdom; sampling rate 50 Hz, dimensions 0.043 x 0.040 x 0.013 m, MEMS accelerometer ± 8g). Physical activity will be computed for 6 days of normal activity and during the 24 hours before the start of each stress test (Fig 3). Recorded accelerometer data will be processed in RStudio (Version 1.2.5033; RStudio Team (2019). RStudio: Integrated Development for R. RStudio, Inc., Boston, MA URL http://www.rstudio.com/) using the GGIR package (Version 2.3–0; https://cran.r-project.org/web/packages/GGIR/index.html) [48].

#### Walking stress test

Participants will be asked to refrain from sports or vigorous physical activities (e.g. gardening, construction work, house moving) for at least 24 hours before the start of each stress test and not to consume food or any other beverages other than water for 1 hour before the start of the stress test. For each participant, the start of the stress test will be scheduled at the same time of day with at least one day of rest between stress test days.

At the start of the stress test, participants will rest for 60 minutes before the first blood sample t_pre_ will be taken. Immediately thereafter, they will walk for 30 minutes on the treadmill at self selected walking speed (recorded on baseline day I) followed immediately by another blood sample (t_post_). Participants will then rest for 3.5 hours, and blood samples will be taken at 30 minutes (t_post+30_), 120 minutes (t_post+120_) and 210 minutes (t_post+210_) after the end of the walking stress (Fig 3). Participants will be allowed to consume a standardized snack immediately after blood sample t_post+120_.

**Fig 5:**
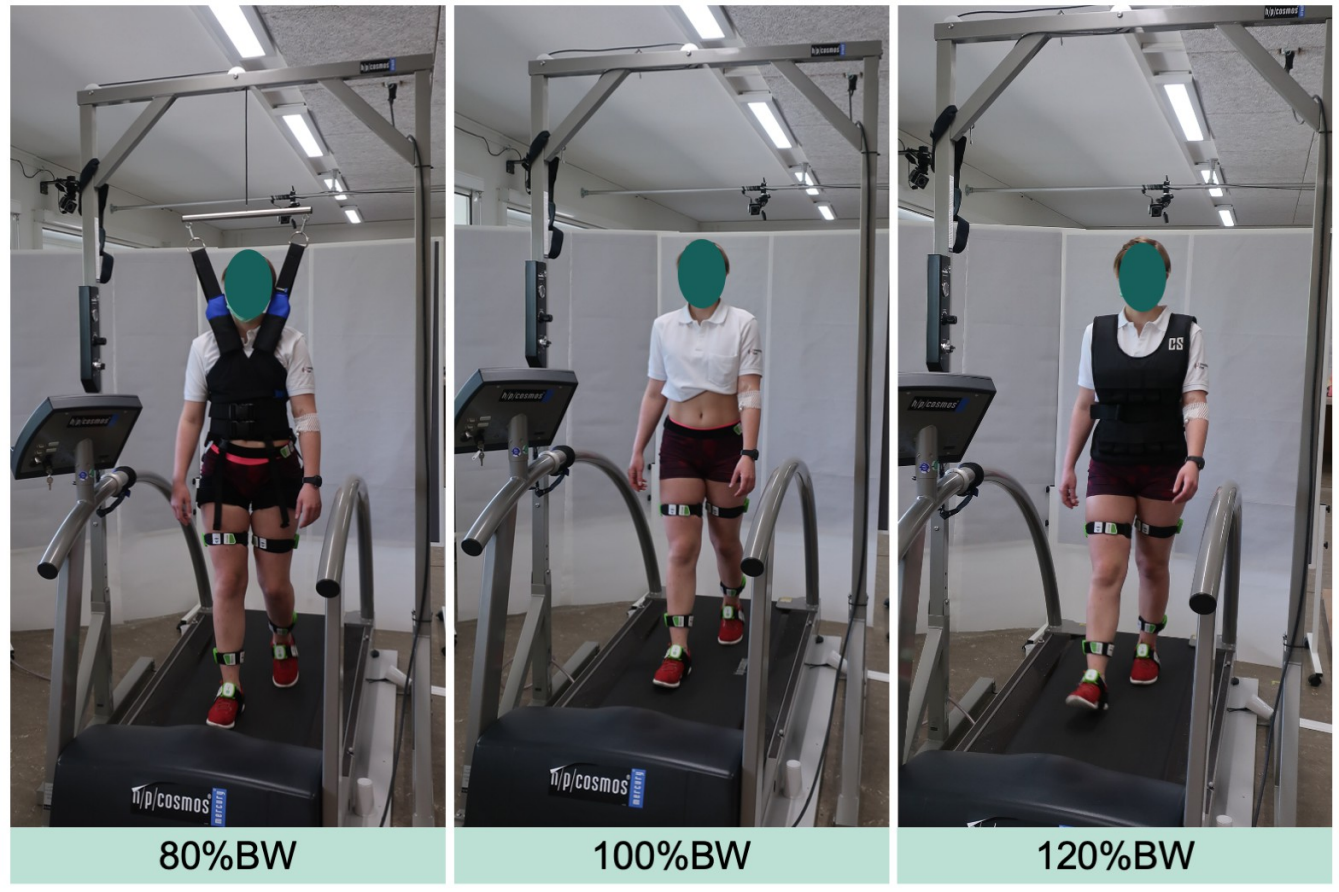
The applied loading conditions during walking stress. Reduced load (80% bodyweight (BW)) is achieved using a harness connected to a pneumatic pulley system (left). During normal load (100%BW) the BW is not altered (middle). Increased load (120%BW) is achieved using a weight vest (right).

In each of the stress tests, either one of the three loading conditions 80%BW, 100%BW or 120%BW will be applied [32–34]. The reduced load condition will be achieved via an unloading system comprising a steel frame and a pneumatic pulley system connected to an unloading harness (h/p/cosmos airwalk®, h/p/cosmos sports & medical GmbH, Nussdorf-Traunstein, Germany). The increased load condition will be achieved via weight vest with equal weights applied to the front and back (1 kg increments). With this experimental framework, the participant’s BW is dynamically altered to 80%BW and 120%BW, respectively, throughout the entire 30-minute walking stress (Fig 5) [32]. The number of steps will be recorded during the entire stress test using an AchtiGraph activity monitor (ActiGraph wGT3X-BT, Actigraph, Pensacola, FL, USA).

#### Biomechanical assessment during the 30-minute walking stress

During minutes 2, 14, and 27 of the walking stress, spatiotemporal parameters and vertical vGRF will be measured using the pressure plate built into the treadmill (Zebris FDM-THM-S pressure plate, Zebris Medical GmbH, Isny, Germany) and sagittal joint kinematic patterns will be recorded with the inertial sensor gait analysis system RehaGait® (Hasomed, GmbH, Magdeburg Germany) with seven sensors placed on the sacrum, and bilaterally on the lateral thigh, lateral shank, and foot (Fig 5; for more details see [32]).

#### Heart rate measurements during the 30-minute walking stress

Heart rate throughout the entire stress test will be recorded using a heart rate monitor (Polar M430, Polar Electro Europe AG, Steinhausen, Switzerland) with a chest sensor (Polar H10, Polar Electro Europe AG, Steinhausen, Switzerland). Mean heart rate will be extracted using the manufacturers software Polar Flow (https://flow.polar.com/).

#### Biological assessments

Venous blood samples (7.5 ml) will be obtained from the same antecubital vein. A vein catheter (Vasofix® Safety PUR 20G, B. Braun Melsungen AG, Melsungen Germany) will be placed before tpre and stay in place for the rest of the entire stress test (4 hours; Fig 3). After each blood draw, the catheter will be flushed with 5 ml isotonic saline solution (0.9% NaCl) to prevent plugging through clotting blood. The first 3 ml of each sample will be discarded to avoid dilution through the prior injected saline solution. The blood samples will clot in the blood tubes (S-Monovette ® 7.5 ml Z-Gel, Sarstedt AG, Nürnbrecht, Germany) for 30 minutes, be centrifuged (Sarstedt AG & Co SMC6) for 15 minutes at 2016g, be stored in the fridge (4°C) for less than 2 hours until separation in aliquots, and frozen (-80°C). The blood sample at the 24-month follow-up (t_24_) will be taken immediately after the MRI using a needle (Venofix® Safety 21G, B. Braun Melsungen AG, Melsungen Germany) and the same blood tube and blood processing as for the baseline data collection samples. All blood samples will be stored in a biobank allowing for additional future analyses beyond those specified here.

#### Assessing serum biomarker concentrations

The concentration of biomarkers sCOMP, sMMP-3 and sIL-6 will be determined using commercially available enzyme-linked immunosorbent assays (ELISA). Investigators will be blinded to the samples, which will be analyzed in duplicates and in random order. Differences due to inter-assay variation will be eliminated by comparing concentrations within participants and testing all samples of any participant on the same plate. All blood analysis will be carried out by a service provider who will not receive any information on the samples other than the participant code and random sample numbers to ensure that all samples of the same participant will be placed on the same plate.

### Data management

All study data will be entered into and managed using REDCap (Research Electronic Data Capture) hosted at our orthopaedics clinic [49,50]. REDCap is a secure, web-based software platform designed to support data capture for research studies.

### Statistical analysis

Statistical analyses will be performed using RStudio Team (2019; Version 1.2.5033). For Specific Aim 1, the primary outcome parameter will be concentration of sCOMP, sMMP-3 and sIL-6 at the five timepoints, and the secondary outcome parameter will be ambulatory load (vGRF), age and tissue health (T2 relaxation time, cartilage thickness; Table 2). Based on a visualization of the biomarker concentration distribution and the typical biomarker kinetics over time of the stress test, we will define outcome parameters reflecting the response to the stress test (e.g., initial change t_post_ compared to t_pre_ and the delayed change back to the initial level). Further we will use systemic inflammation status (IL-6 concentration at t_pre_) as covariate in subsequent analyses. We will investigate the linearity of change in outcomes dependent on ambulatory load magnitude (slope), and variance heterogeneity of the outcomes dependent on ambulatory load magnitude, age, tissue health and systemic inflammation status (IL-6 concentration at t_pre_). The sensitivity of each biomarker to ambulatory load magnitude stratified by age, tissue health and inflammation will be tested in mixed models. Further explanatory and hypotheses generating analyses are planned with respect to new definitions of potential markers.

**Table 2:** Primary and secondary outcome parameters for Specific Aims 1 and 2.

	*Primary outcome parameter*	*Secondary outcome parameter*	*Additional outcome parameters*
*Specific Aim 1*	• Concentration of sCOMP, sMMP-3 and sIL-6 at five timepoints of the three stress tests	• Ambulatory load (vGRF) • Age • Tissue health (T2 relaxation time, cartilage thickness)	• Physical activity level during 24 hours prior to the stress test • Heart rate during walking stress • Sagittal joint kinematics during the walking stress • Additional blood biomarkers • Baseline KOOS, KSS
*Specific Aim 2*	• Change in tissue health (T2 relaxation time, cartilage thickness) between baseline and 24-month follow-up	• Baseline slope of serum biomarker response • Baseline kinematic and kinetic gait parameters • Change in cartilage thickness • Change in KSS and KOOS • Change in physical activity level	• Concentration of serum biomarker at 24-month follow-up

sCOMP–serum cartilage oligomeric matrix protein; sMMP-3 –serum matrix metalloproteinase-3; sIL-6 –serum interleukin-6; vGRF–vertical ground reaction force; KSS–Knee Society Score; KOOS–Knee Injury and Osteoarthritis Outcome Score.

For Specific Aim 2, additional clinical outcomes measured both a baseline and at the 24-month follow-up data collection will be used. The primary outcome parameter will be the change in T2 relaxation time and cartilage thickness. Secondary outcome parameters will be the slope of serum biomarker response taken from the analysis of Specific Aim 1, kinematic and kinetic gait parameters, the change in KOOS score, modified KSS score and physical activity levels. The distribution of the outcomes will be depicted in an initial analysis (Table 2). The research questions will be addressed by testing the association of the slope with the difference in T2 relaxation times and cartilage thickness using regression models. Further we will investigate the independent prognostic value of the individual slopes for each biomarker by corresponding adjustments, first for age and then also for tissue health and inflammation. We will investigate a potential effect modification by physical activity by adding corresponding interactions terms to the regression models. In all regression models, the baseline value of the clinical outcome will be included as covariate. Since the individual slopes are estimated and we can assess their standard error, we will correct for this measurement error using regression calibration. The significance level for all statistical tests will be set a priori to 0.05.

#### Sample size calculation

To judge the adequacy of the intended samples sizes, we performed simulation studies using the estimated population means, the estimated populations standard deviations [33,34] and the estimated residual variance as true parameter values and assuming a correlation of 0.8 between intercept and slope. We then varied the population standard deviation of the slope by factors of 2 and 3 because in this study we expect a substantially larger population variation due to wider age range and including both ACL injured and healthy participants. We conducted three simulation studies corresponding to the intended main analyses based on the planned sample size of 96 participants and including 2500 repetitions per simulation.

For Specific Aim 1, the first simulation study looked at the standard error of the estimates of the population standard deviation of the slope. We observed standard errors of 1.87, 1.27, and 1.24 for population standard deviations of the slope of 3.7, 7.4 and 11.1, respectively, suggesting that we can obtain a rather precise picture of the population variation. In the second simulation, we looked at the power to demonstrate a significant interaction between load and a single normally distributed covariate that can explain some of the biological variation of the slope. Already for a population standard deviation of 3.7, we obtained a power of 71% if the covariate can explain 50% of the biological variation. For a population standard deviation of 7.4, we reached a power of 85% if the covariate can explain at least 25% of the variation. For a population standard deviation of 11.1, we obtained a power of 89% if the covariate can explain at least 20% of the variation. In the third simulation, we reached a power of 70% if the covariates explain together at least 82% of the variation, and for a population standard deviation of 11.1 we reach a power of 80% if they explain together 74% of the variation for a population standard deviation of 7.4. This suggests that we will be able to demonstrate interactions between tissue health / inflammation status and ambulatory load on top of the interaction with age if the interactions are of similar magnitude as those for age, if all three factors explain a substantial amount of the biological variation, and if we have a substantial population variation of the slope.

For Specific Aim 2, we have a power of 72% to find a significant association if the true correlation is 0.3, and a power of 95% if the true correlation is 0.4, taking into account a potential drop-out rate of 10%. With respect to the ability of assessing an independent prognostic value, we performed a simulation study where age, tissue health and inflammation can explain 40% of the variation in the individual slopes and assuming that these three variables together can explain the variation in the clinical outcome to the same degree as the individual slopes. We obtain a power of 80% to demonstrate an independent prognostic value if the individual slopes have a partial correlation of 0.6 with the outcome.

## Discussion

In this study, we will focus on the factors age, tissue health, and inflammation, and on specific biomarkers for articular cartilage. Age is the most prominent factor associated with the development of knee OA [1], and the risk for OA increases after joint injuries [51] possibly triggered or exaggerated by an inflammatory response after injury that may be reinitiated after ACL reconstruction [52].

In a previous study, all KOOS subscores were lower than in age, sex and sports matched uninjured participants 3 to 10 years after intra-articular knee joint injury in 15 to 26 year old participants [53]. Twelve years after ACL injury, 34 of 67 female football players (mean age of 31 years) had radiographic patellofemoral or tibiofemoral OA in their ACL injured knee but only 5 of 65 players had radiographic tibiofemoral OA in their contralateral knees (3 also ACL injured, 1 meniscus injury, 1 unknown injury) [54]. A recent umbrella systematic review reported an almost tenfold greater risk for knee osteoarthritis 10 years after ACL injury compared to uninjured persons with a slightly greater risk in those who were treated surgically [5]. Based on this umbrella review, we decided to include participants with prior ACL rupture independent of treatment to increase the variability in tissue health and inflammation [5].

The mechanisms involved in the development of post-traumatic OA have been discussed in the literature [55–58]. Recently, a multifactorial model for post-traumatic OA after ACL injury was introduced, where they distinguish between structural factors (cartilage, meniscus, subchondral bone), biological factors, neuromuscular factors and mechanical factors that are altered with and after ACL injury [58]. Based on this evidence, we expect that persons 2 to 10 years after ACL injury independent on the type of treatment received (operative or conservative) will have a high risk of presenting with compromised tissue health that may be termed early OA.

COMP is a structural protein found in articular cartilage, tendon, meniscus or ligaments [59] that binds to collagen I/II and IX, plays an important role in the organization and maintenance of the ECM [60,61], is involved in fibrillogenesis [62], and is believed to be involved in the activation of mechanisms that protect and repair ECM [63]. sCOMP concentration is one of the most studied articular cartilage blood biomarkers [64] with higher sCOMP in knee OA patients compared to controls [65–67], and higher levels with greater OA severity [65] and number of joints affected by OA [68]. After ACL injury, resting sCOMP concentration was higher than in an age, sex and activity level matched control group [69]. In healthy persons, sCOMP increased temporarily after walking [70–72] and decreased during immobilization [73–75]. Furthermore, post walking stress sCOMP levels correlated with long-term changes in cartilage thickness in the medial femur [76] and tibia [76,77].

MMP-3 is an enzyme that degrades different components of the ECM proteins [78], has the ability to bind to triple helical regions of collagens [79,80], activates procollagenase activity [81] and thereby regulates collagen turnover [79]. In a meta-analysis, it was reported that the standardized mean difference for sMMP-3 levels did not differ between patients with knee OA and controls [67]. In a recent study, participants with knee OA had higher sMMP-3 levels than controls [66], and among participants with knee OA those with progression of cartilage injury in the medial knee compartment had higher levels of sMMP-3 [82]. In healthy participants sMMP-3 levels decreased during immobilization [74], were temporarily increased after walking and this load-induced increase was dependent on ambulatory load magnitude [34].

The proinflammatory cytokine IL-6 activates the immune system and increases the inflammatory response [83]. sIL-6 was significantly higher in participants with radiographic confirmed knee OA [84], predicted the loss of tibial cartilage loss measured in MRI’s over 3 years [85] and was associated with lateral tibial cartilage volume loss over 11 years [86]. Further, it is also believed that IL-6 increases the expression of anti-catabolic factors and thereby has a protective role [87]. Because IL-6 is involved in anabolic and catabolic pathways, it remains unclear if and how sIL-6 concentrations affect the mechanoresponse of cartilage blood biomarkers. sIL-6 concentration may be affected by other pathological conditions such as viral infections [88] and these altered systemic sIL-6 concentrations may also affect the load induced mechanoresponse of articular cartilage blood markers.

Based on a recent comprehensive analysis of 10 blood biomarkers [34], sCOMP, sMMP-3 and sIL-6 were among the candidates with a potential for investigating the role of articular cartilage mechanosensitivity in knee OA.

The results of this study will provide first evidence of the role of age, tissue health or presence of inflammation for the dose-response relationship between ambulatory load magnitude and load-induced changes in mechanosensitive biomarkers of articular cartilage and if this relationship may be used as predictor of future articular cartilage degeneration. The blood biomarkers of articular cartilage included in this study are critical players in the regulation of cartilage metabolism in health and disease. Confirming a dose-response relationship of ambulatory load magnitude and changes in systemic blood biomarkers of articular cartilage and modulating effects of inflammation, age and tissue statues will clearly show their importance for in vivo mechanobiology of articular cartilage. Blinding participants to the experimental condition is not possible because of the obvious differences between conditions (reduced load and increased load). However, the person processing the data will be blinded to the condition. Because it does not seem feasible that a subject can actively alter the load-induced changes in blood markers of articular cartilage, it is assumed that this approach is appropriate for answering the research questions.

Revealing a role of this relationship in cartilage degeneration will be the basis for using these biomarkers as potential targets for pharmacologic agents and load-modifying interventions aimed at changing tissue metabolism in the context of OA pathomechanics that can be further investigated in ex vivo, in situ and in animal models of OA. Future analyses using emerging technologies (such as proteomics) of the samples obtained here will be possible as all samples will be stored in a biobank. Moreover, current research efforts at our institution [89] go into manufacturing cartilaginous grafts with nasal chondrocytes embedded in an ECM rich in glycosaminoglycan and type II collagen and these tissues have been successfully secured in the injured joints in patients. It will be of high scientific interest to use the methods described in this study protocol to study these engineered tissue grafts in the living joint because the prognosis of these grafts and the surrounding native tissue is currently unknown and will likely depend on the in vivo load applied to these grafts. In addition, because the mechanosensitivity of articular cartilage may reflect tissue health, the presented novel study protocol may represent a diagnostic test for early OA. Finally, with using this study protocol, ambulatory joint motion and load could be routinely assessed as parameters for therapy planning and evaluation of orthopaedic interventions and be used as outcome measures of clinical trials investigating orthopaedic interventions.

The results of the proposed study will extend current knowledge of the influence of ambulatory load on articular cartilage blood biomarker response and will thereby extend current knowledge on in vivo biomarker metabolism. This may lead to the identification of new therapeutic intervention strategies (surgical, physiotherapeutic or pharmacologic). Moreover, the results of the long-term data collection will help to evaluate the prognostic value of the load induced cartilage biomarker response for articular cartilage degeneration, which helps to identify people at risk or with a fast progression of cartilage degeneration.

## Supporting information

S1 File SPIRIT checklist. (PDF)

S2 File First approved study protocol by EKNZ. (PDF)

S3 File First approval of study protocol by EKNZ. (PDF)

S4 File Last amendment of study protocol to EKNZ. (PDF)

S5 File Approval of last amendment of study protocol by EKNZ. (PDF)

S6 File Letter of funder. (PDF)
