# Supplementary material for: Dose-response relationship of in vivo ambulatory load and mechanosensitive cartilage biomarkers—The role of age, tissue health and inflammation: A study protocol
Source: PLoS One. 2022 Aug 19;17(8):e0272694. doi: 10.1371/journal.pone.0272694 (PMC9390933; doi:10.1371/journal.pone.0272694)
Supplement: S2 File — (PDF) [file pone.0272694.s002.pdf]

Project Title

MechSens – Dose-response relationship of in vivo ambulatory load and mechanosensitive cartilage biomarkers: the role of age and tissue health

Research legislation:	Ordinance on human research with the exception of Clinical trials[1] (HRO).
Type of Research Project:	Research project involving human subjects
Risk Categorisation:	Risk Category A
Project Leader:	Prof. Dr. Annegret Mündermann, Head Functional Biomechanics, Department of Orthopaedics and Traumatology, University Hospital Basel, Spitalstrasse 21, 4031 Basel, Tel. 061 328 5445, Fax 061 265 26 50, Email annegret.muendermann@unibas.ch.

PROTOCOL SIGNATURE FORM

Study Title	*Dose-response relationship of in vivo ambulatory load and mechanosensitive cartilage biomarkers: the role of age and tissue health*

The project leader has approved the protocol version 2, (13.08.2019) and confirms hereby to conduct the project according to the protocol, the Swiss legal requirements[1,2], current version of the World Medical Association Declaration of Helsinki[3] and the principles of Good Clinical Practice.

Project leader:

Prof. Dr. Annegret Mündermann Ph.D.
Department of Orthopaedics and Traumatology, University Hospital Basel, Spitalstrasse 21, 4031 Basel

Date: _________________________ Signature: ____________________________

TABLE OF CONTENTS

TABLE OF CONTENTS		3
GLOSSARY OF ABBREVATIONS		4
1	BACKGROUND AND PROJECT RATIONALE	5
2	PROJECT OBJECTIVES AND DESIGN	6
	2.1 Hypothesis, primary and secondary objective	6
	2.2 Primary and secondary endpoints	7
	2.3 Project design	8
3	PROJECT POPULATION AND STUDY PROCEDURES	8
	3.1 Project population, inclusion and exclusion criteria	8
	3.2 Recruitment, screening and informed consent procedure	9
	3.3 Study procedures	9
	3.4 Withdrawal and discontinuation	14
4	STATISTICS AND METHODOLOGY	14
	4.1. Statistical analysis plan	14
	4.2. Handling of missing data	17
5	REGULATORY ASPECTS AND SAFETY	17
	5.1 Local regulations / Declaration of Helsinki	17
	5.2 Notification of safety and protective measures (HRO Art. 20)	18
	5.3 Serious events (HRO Art. 21)	18
	5.4 Amendments	18
	5.5 End of project	18
	5.6 Insurance	18
6	FURTHER ASPECTS	18
	6.1 Overall ethical considerations	18
	6.2 Risk-Benefit Assessment	18
7	QUALITY CONTROL AND DATA PROTECTION	19
	7.1 Quality measures	19
	7.2 Data recording and source data	19
	7.3 Confidentiality and coding	19
	7.4 Retention and destruction of study data and biological material	20
8	FUNDING / PUBLICATION / DECLARATION OF INTEREST	20
9	REFERENCES	20

GLOSSARY OF ABBREVATIONS

ACL	*Anterior cruciate ligament*
ADMTS	*A disintegrin and metalloproteinase with thrombospondin motifs*
BASEC	*Business Administration System for Ethical Committees*
BMI	*Body mass index*
BW	Bodyweight
COMP	*Cartilage oligomeric matrix protein*
CPII	*C-propeptide*
CRF	*Case report form*
C2C	*COL2−3/4Clong mono epitope*
DESS	*Double-echo in steady-state*
ECM	*Extra cellular matrix*
EMG	*Electromyography*
FA	*Flip angle*
FOPH	*Federal Office of Public Health*
FOV	*Field of view*
GRF	*Ground reaction force*
HRA	*Human Research Act*
HRO	*Ordinance on Human*
IL-6	*Interleukin-6*
KOOS	*Knee Injury and Osteoarthritis Outcome Score*
KSS	*Knee Society Score*
MMP	*Matrix metallopeptidase*
MRI	*Magnetic resonance imaging*
OA	*Osteoarthritis*
PA	*Physical activity*
PRG	*Proteoglycan*
PS	*Pixel size*
ST	*Slice thickness*
TE S +	*Echo time S+*
TE S -	*Echo time S-*
TR	*Repetition time*

1 BACKGROUND AND PROJECT RATIONALE

The WHO estimated that more than 10% of people older than 60 years suffer from osteoarthritis (OA). OA is a degenerative joint disease that affects different joints with symptoms like joint pain, limitation in movement, crepitus or local inflammations.[4] Pathogenic changes in the articular cartilages structure happen long before symptoms are recognized.[5] Structural changes in the late OA stages (joint space narrowing and osteophytes) in osteoarthritic joints can be confirmed radiologically[6] or using magnetic resonance imaging (MRI). Today, MRI is used most commonly because it has the ability to visualize damage in cartilage and other joint tissues as well as bone marrow and meniscal lesions.[7,8] MRI parameters include cartilage thickness, volume and quality assessed with new and valid MRI[9] double-echo in steady-state (DESS), which is promising for detecting even early-stages of OA.[10]

Articular cartilage is avascular and aneural, and transport of nutrients and waste products in and out of cartilage occurs through diffusion. Under normal physiologic conditions, chondrocytes (the cells in cartilage) synthesize and maintain crucial extracellular matrix (ECM) components that confer the functional properties of cartilage.[11] Main components of the ECM are water, collagens (90% to 95% Type II), sulfated proteoglycans and non-collagenous proteins.[12-14] Under pathologic conditions such as OA, chondrocytes exhibit an imbalance of anabolic and catabolic activities that are characterized by degenerative changes in the cartilage matrix and other joint tissues, including the subchondral bone and synovium.[15-17] Although the molecular mechanisms that trigger the pathological changes in the initiation of OA are largely unknown, the ability of chondrocytes to respond to load is believed to play a critical role for maintaining healthy tissue and in the initiation of OA. To extend the current knowledge about articular cartilage we investigate blood biomarkers as surrogates for the articular cartilage's metabolism. Candidates for mechanosensitive blood markers of articular cartilage include structural proteins, cytokines and enzymes involved in tissue metabolism.

In a pilot study, we established an experimental framework to modulate ambulatory load and showed that the increase of concentration of cartilage oligomeric matrix protein (COMP)[18] and other blood markers for articular cartilage health[19] depends on the magnitude of the applied load. Other mechanosensitive markers are matrix metallopeptidase (MMP)-3, MMP-9[20], proteoglycan 4 (PRG-4), A disintegrin and metalloproteinase with thrombospondin motifs (ADAMTS) -4 and ADAMTS-5[21-23], type II procollagen (CPII)[24], and fragments such as the COL2−3/4Clong mono epitope (C2C)[25]. Further, after joint injury, proinflammatory cytokines are elevated and lubricin synthesis decreases predisposing articular cartilage to degeneration and OA onset via changes in joint friction.[26]

The current state of research in the field and of our own research clearly shows that the dose-response relationship between ambulatory load and mechanosensitive blood markers of articular cartilage is poorly understood. Results of ex vivo and in situ studies and of our in vivo studies on changes in levels of blood markers of articular cartilage after a walking stress test raise the following questions:

1. Is there a biological variation in the dose-response relationship between ambulatory load and mechanosensitive blood markers of articular cartilage and to which degree can it be explained by age, tissue status or the presence of inflammation?
2. Does the individual dose-response relationship between ambulatory load and mechanosensitive blood markers of articular cartilage predict future cartilage degeneration in persons at risk for developing early OA?

Clearly, there are multiple factors including mechanical and biological factors that are important to consider. Answering these questions will provide information that help to sort out the processes underlying the in vivo mechanosensitivity of articular cartilage and its potential role in the initiation of knee OA.

This study can be considered as proof-of-concept of a potential diagnostic test (walking stress test) for cartilage mechanosensitivity and will provide first evidence of the role of age, tissue status and presence of inflammation on the dose-response relationship between in vivo ambulatory load and mechanosensitive blood markers of articular cartilage and its relevance for prognosing cartilage degeneration. These results will allow to judge the importance of mechanosensitive blood markers for in vivo mechanobiology of articular cartilage. The results of this study will reveal if the proposed experimental framework may be suitable in the area of cartilage engineering and transplantation and for testing pharmacologic agents and load-modifying interventions aimed at changing tissue metabolism in the context of OA pathomechanics that can be further investigated in ex vivo, in situ and in animal models of OA.

This project is a risk category A project. Collecting health-related personal data and biological samples in this study entail minimal risks and burdens for the patient.

2 PROJECT OBJECTIVES AND DESIGN

2.1 Hypothesis, primary and secondary objective

This project will test the overall hypothesis that there is biological variation in the relationship between ambulatory load magnitude and mechanosensitive blood markers of articular cartilage, that this variation can be partially explained by a dependence on age, tissue status and inflammation representing an in vivo model for assessing cartilage mechanosensitivity, and that this variation predict early articular cartilage degeneration.

The primary objective is to investigate the in vivo dose-response relationship of weight bearing and mechanosensitive blood markers of articular cartilage using controlled weight bearing during a walking stress test and age, tissue status and the presence of inflammation as experimental paradigms.

- **Hypothesis 1.1:** The slope of the relationship between ambulatory load and mechanosensitive blood markers of articular cartilage will be smaller in older subjects than in younger subjects.
- **Hypothesis 1.2:** The slope of the relationship between ambulatory load and mechanosensitive blood markers of articular cartilage will be smaller in subjects with low cartilage quality than in healthy subjects.
- **Hypothesis 1.3:** The slope of the relationship between ambulatory load and mechanosensitive blood markers of articular cartilage will be smaller in subjects with signs of inflammation than in healthy subjects.

We expect that with age or after injury, mechanosensitivity may be altered because the load applied to the chondrocytes is changed due to a disrupted ECM (change in mechanoenvironment of chondrocytes) or age-associated changes in material properties, or because of biological changes such as inflammation or with aging.

The secondary objective is to investigate the prognostic ability of the individual in vivo dose-response relationship of ambulatory load and mechanosensitive blood markers of articular cartilage for articular cartilage degeneration.

- **Hypothesis 2.1:** The slope of the relationship between ambulatory load and mechanosensitive blood markers of articular cartilage negatively correlates with subsequent decrease in articular cartilage quality.
- **Hypothesis 2.2:** The slope of the relationship between ambulatory load and mechanosensitive blood markers of articular cartilage negatively correlates with subsequent articular cartilage thinning within 2 years.

- **Hypothesis 2.3:** The slope of the relationship between ambulatory load and mechanosensitive blood markers of articular cartilage correlates with the change in Knee Injury and Osteoarthritis Outcome Score (KOOS) and modified Knee Society Score (KSS).
- **Hypothesis 2.4:** Weekly physical activity levels influence the associations postulated in Hypotheses 2.1, 2.2 and 2.3.

We expect that altered mechanosensitivity will lead to articular cartilage degeneration measured as articular cartilage thinning and decrease in articular cartilage quality and that physical activity level modulates this relationship. Other potential influencing factors in patients with anterior cruciate ligament (ACL) injury include treatment (conservative or operative), time of operation (if applicable), duration of physiotherapy, time of return to work, time of return to sport, or type of sports before injury.

2.2 Primary and secondary endpoints

In the proposed study, we will use a previously tested experimental approach[18] involving modulating the magnitude of in vivo ambulatory load during a walking stress test. We have previously identified candidate blood markers[19] with the highest sensitivity to ambulatory load magnitude and highest biological variation of the relationship between load-induced changes in blood levels and ambulatory load. We will use the same experimental approach in a new cohort to study the effects of age, tissue status and the presence of inflammation on the in vivo dose-response relationship of ambulatory load and mechanosensitive blood markers of articular cartilage using complex statistical models. Further, we will relate the individual dose-response relationship between ambulatory load magnitude and mechanosensitive blood markers of articular cartilage to prospectively measured changes in articular cartilage thickness and quality while considering the potential influence of physical activity level.

Primary endpoints

- Slope of the relationship between ambulatory load and mechanosensitive blood markers of articular cartilage.

Secondary endpoints

- Change in T2 relaxation time and cartilage thickness from baseline to follow-up
- Score in questionnaires: Modified KSS and the KOOS
- Change in modified KSS and KOOS scores within the 2 years
- Age, bodyweight (BW) sex of the subject
- Inflammation status at baseline (IL-6)
- Physical activity (PA) level during 7 days after the baseline (prior to the experiment), during the experiment and during 7 days after the follow-up measurement.
- Ambulatory load at each condition (maximum vertical ground reaction force (GRF) and total vertical ground reaction impulse during walking)
- Number of steps taken during the test day
- Joint kinematics for the three loading conditions (dynamic range of motion during walking at the ankle, knee and hip)
- Joint kinematics for the normal load condition (rotational offset and difference in maximum knee moment between injured and not injured knee)
- Heart rate during and after the walking stress test
- Tissue status at baseline: T2 relaxation time and cartilage thickness

Other parameters

Treatment (conservative or operative), time since injury, time of operation (if applicable), duration of physiotherapy, time of return to work, time of return to sport, type of sports before injury.

2.3 Project design

Prospective experimental multimodal (clinical, biomechanical, biological) data collection with block randomization and cross-over.

We will recruit equal numbers of male and female subjects in each group. Conditions will be block randomized in two blocks of possible condition orders per sex per subject group to prevent a potential systematic condition effect. Blinding to the experimental condition is not possible because of the obvious differences between conditions (partial weight bearing and additional load). However, the person processing the data will be blinded to the condition. Because it does not seem feasible that a subject can actively alter the load-induced changes in blood markers of articular cartilage, it is assumed that this approach is appropriate for answering the research questions.

3 PROJECT POPULATION AND STUDY PROCEDURES

3.1 Project population, inclusion and exclusion criteria

We will examine a cohort of 96 subjects. Experiments for all sub aims will involve the same subjects (N=24 per group):

- Group 1: healthy subjects aged between 20 and 30 years
- Group 2: subjects with previous ACL injury aged between 20 and 30 years
- Group 3: healthy subjects aged between 40 and 60 years
- Group 4: subjects with previous ACL injury aged between 40 and 60 years

A minimum age of 20 years and maximum age of 60 years was chose to ensure skeletal maturity and to include an age range with increased risk of early knee OA (above 40 years[27]).

Inclusion criteria for group 1 and 3

- Being physically active (>2hours/week)
- No previous known knee injury

Inclusion criteria for group 2 and 4

- Being physically active (>2hours/week)
- ACL rupture between 2 to 10 years prior to the study

General exclusion criteria

- Inability to provide informed consent
- Age < 20 years (before maturation) or age > 60 years
    - Advanced general sarcopenia (degenerative loss of muscle mass in aging) and high likelihood of osteoarthritic changes
- Body mass index (BMI) > 35 kg/m^2:
    - Excessive skin movement that influences the gait analysis
    - Inability to walk for 30 minutes
- Contraindications for a knee MRI
- Active rheumatic disorder
- Prior neuromuscular impairment (e.g. stroke)
- Conditions other than knee injury that could cause abnormal patterns of locomotion

- Prior hip, knee, and ankle prosthesis
- Osteotomy of the lower extremities
- Prior spine surgery
- Other major medical problems
- Pregnancy
- Investigators and their immediate families are not permitted to be subjects
- Persons who have previously completed or withdrawn from this study
- Patients currently enrolled in another experimental (interventional) protocol

3.2 Recruitment, screening and informed consent procedure

Healthy subjects and subjects with ACL injury will be recruited from the community surrounding the University Hospital Basel by placing a flyer in the newspaper, the institutional website, social media, and distributing flyers at local orthopaedic and physiotherapy clinics, sports clubs and the University campus. Advertisements will be placed bi-monthly in the University of Basel "Uni-news" and ongoing on the department website. This flyer contains the core research goal and the contact information of the project manager. Additional we will review the patient list of the University Hospital Basel for people with ACL injuries 2 to 10 years ago. Eligible candidates will be contacted by telephone and asked if they want to receive further information for this study. Interested persons will be contacted by the project manager via telephone or email. After the first telephone contact with the study personnel, the patient informed consent form will be sent to the volunteers (usually at least 4 weeks before the data collection). Volunteers will be given about 1 week to make a final decision to participate. Any questions regarding the study will be directed to and answered by the project manager. One week after the initial contact the project manager will inquire whether the volunteer is interested in participating in this study or not. Upon agreement to participate, the appointments for the baseline day and the three test days will be arranged by telephone. Experience from previous studies on healthy subjects and patients with orthopaedic conditions have shown that this recruitment strategy is successful. Before any examination starts, the participant and the project manager will go through the participant information document ensuring informed consent. A copy of the signed informed consent will be given to the study subject. The consent form will be retained as part of the study records.

We will provide a small financial compensation (200CHF per subject: 100CHF after the baseline assessment and 100CHF at the 24-month follow-up) and a summary of their ambulatory gait function as appreciation for their time commitment.

The study population will not comprise any vulnerable participants.

3.3 Study procedures

Each subject participating in this study undergoes a recruitment and information process that will last over approximately 1 month. The data collection (baseline day 1 and test days I, II and III) will happen on 4 different days within 1 month and with at least 1-day break between the test days (Figure 1). The follow-up will be scheduled 24 months after the baseline measurement (Figure 2). On the baseline day, the participant will complete two questionnaires (KSS, KOOS) concerning the health of their knees. Further, subjects will be clinically assessed by an orthopaedic surgeon, participants with childbearing potential will undergo a pregnancy test, and an MRI of both knees and gait data at least 7 days prior to the experiment will be recorded. Tissue status will be determined by the T2 relaxation time and thickness of weight bearing knee cartilage. Participants will be asked to wear an activity monitor (ActiGraph GT3X+, Pensacola, FL, USA) for 7 days prior to the first test day.

Each participant will complete the walking stress test with repeated blood sampling on three test days with at least 24 hours without physical activity before each test day (Figure 1; always at the same time of the day). On each test day, participants will walk for 30 minutes on a treadmill with

either one of the three loading conditions (reduced load = 80% BW, normal load = 100% BW, increased load = 120% BW). The order of experimental condition will be applied in randomized order determined by block randomization, and the same self-selected walking speed will be used for all conditions. Subjects will be tested at least 2 hours after waking and will not be allowed to eat 1 hour prior the test and until blood sample t_2 was taken during the walking stress test. They will be allowed to eat a standardized snack after blood sample t_2. During the walking stress test gait kinematics and GRF will be measured with an inertial sensor based gait analysis system (RehaGait ®; Hasomed GmbH, Magdeburg, Germany) and with the pressure plate embedded in the treadmill (h/p/cosmos, Zebris FDM-T, Isny, Germany). Heart rate will be recorded during the 30-minute treadmill walking exercise and for 10 subsequent minutes using a heart rate monitor (Polar m400, Polar Electro Europe AG, Steinhausen, Switzerland) with chest strap (Polar H7, Polar Electro Europe AG, Steinhausen, Switzerland). Subjects will rest in a seated position 1 hour before and 3.5 hours after the walking exercise. Serum levels of mechanosensitive blood markers of articular cartilage will be assessed in all blood samples collected during the experiment (Figure 1).

To answer the secondary objective, participants will be clinically assessed by an orthopaedic surgeon and undergo MRI of both knees 24 months after the baseline assessment (Figure 2). Participants with childbearing potential will undergo a pregnancy test. In addition, subjects will be asked to wear an activity monitor for the 7 days after the follow-up to record the typical level of physical activity. The slope of the relationship between ambulatory load magnitude and load-induced changes in blood markers of articular cartilage will be taken from the primary objective. Cartilage degeneration will be assessed as the difference in T2 relaxation times and cartilage thickness between baseline and 24-month follow-up, respectively. Physical activity level will be assessed as number of steps taken and time spent in moderate to vigorous physical activity. For the primary objective the participants will spend about **3 hours** on the baseline day and **5.5 hours** on test days I to III. To answer the secondary objective, the follow-up examination after 24 months requires another 2 **hours**.

The total project duration is 4 years (see timeline).

Timeline

Task	Year 1				Year 2				Year 3				Year 4			
	Q1	Q2	Q3	Q1	Q1	Q2	Q3	Q4	Q1	Q2	Q3	Q4	Q1	Q2	Q3	Q4
Primary Objective																
Data collection, baseline	X	X	X	X	X											
Data processing blood samples			X		X	X										
Data processing MRI	X	X	X	X	X	X	X									
Evaluating Specific Aim 2				X	X		X	X								
Secondary Objective																
Data collection, follow-up									X	X	X	X	X			
Data processing MRI									X	X	X	X	X	X	X	
Evaluating Specific Aim 2												X	X		X	X
Conference presentations						X				X				X		
Manuscript preparation					X	X			X	X			X	X	X	X

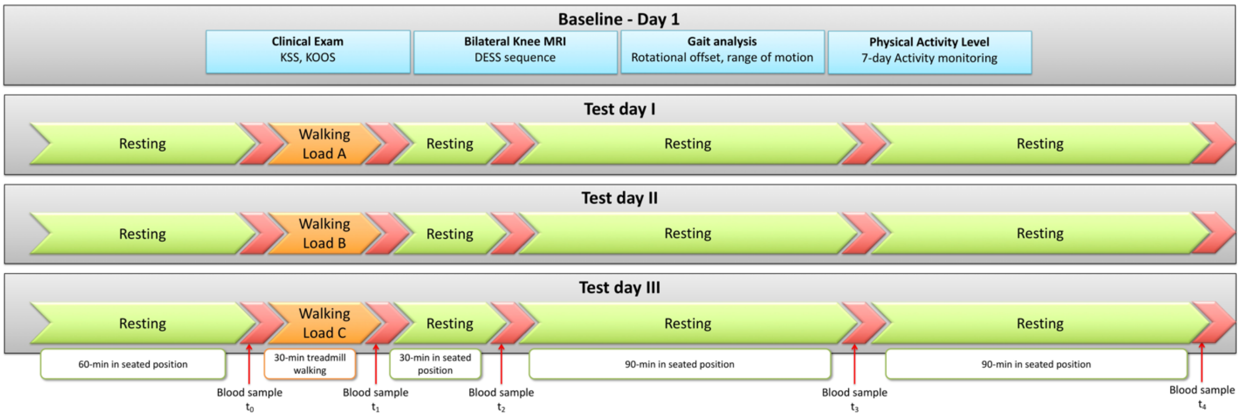

Fig. 1: **Walking stress test protocol with varying ambulatory load.** Three loading conditions (80%, 100% and 120% body weight) will be tested on three separate days (random order). Participants will rest for 1 hour before and 3.5 hours minutes after a 30-minute treadmill walking exercise. Venous blood samples will be taken at five time points.

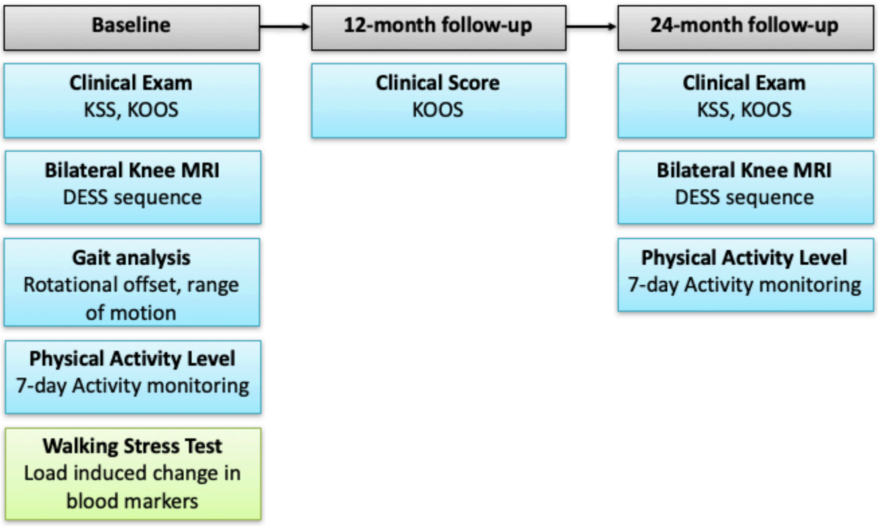

Fig. 2: **Prospective design for assessing cartilage degeneration.** At baseline, a clinical exam, bilateral knee MRI, gait analysis, physical activity level and a walking stress test will be conducted. The dose-response relationship determined in the walking stress test at baseline will be related to changes in articular cartilage thickness and quality from baseline to the 24-month follow-up. The 12-month follow-up serves as additional endpoint to stay in contact with the participants thus minimizing drop-outs.

a) Clinical assessment

The choice of clinical parameters is generally based on the ICHOM standards.[28] Clinical evaluation will be carried out using the modified KSS and its functional component.[29] The KOOS will be used to assess pain, symptoms, activities of daily living, sport and recreation and knee related quality of life.[30]

b) Physical activity level

Physical activity (PA) level during the 7 days prior to the experiment and during the experiment will be recorded using an activity monitor (ActiGraph GT3X+, Pensacola, FL, USA; e.g. number of steps taken, Kcals, PA intensity). The ActiGraph is widely used in scientific research and has been shown to be highly reliable, valid and responsive.[31-33] The ActiGraph will be attached to a waist belt and placed on the subject's right side. Tracking PA levels will allow us to monitor regular PA level and if participants indeed did not perform demanding physical activities on the days prior to test days according to the experimental instructions.

c) Walking stress test

Before the walking stress test, participants will rest for 60 minutes, and blood samples will be taken at the end of the 60-minutes resting time (t_0). They will then walk with either one of the loading conditions for 30-minutes on the treadmill (with the walking speed determined in baseline day) followed immediately by another blood sample (t_1). The inertial sensor based gait analysis system RehaGait ® is attached to the subject's lower extremity to record their joint kinematic patterns during the walking exercise. Subjects will then rest for 3.5 hours, and blood samples will be taken at 30-minutes of the resting time (t_2) and after two additional 90-minute resting intervals (t_3 and t_4; Figure 1). Subjects movements are recorded during the whole test days using the AchtiGraph activitiy monitor. The unloading system comprises a steel frame, weights, a pulley system and an unloading harness (h/p/cosmos airwalk®, h/p/cosmos sports & medical GmbH, Nussdorf-Traunstein, Germany). The loading system consists of an adjustable weight vest in which weights are applied in front and in the back equally. With this system we are able to decrease the participant's BW dynamically to 80% and increase the BW to 120%, for the whole 30-minutes stress test.

d) Modulating ambulatory load

Ambulatory load is modulated using a load modulation framework on an instrumented h/p/cosmos treadmill (h/p/cosmos, Zebris FDM-T, Isny, Germany; 7168 sensors; area, 1.5 * 0.5 m; range, 1–120 N/cm^2; precision, 1-120 N/cm^2 ± 5%; sampling rate, 120 Hz). The magnitude of ambulatory load during the walking stress test will be calculated as GRF impulse times the number of steps taken during the walking stress test. In the reduced load condition, we will reduce ambulatory load using an unloading system comprising a steel frame with an unloading mechanism attached to an instrumented treadmill. Weights corresponding to 20%BW of each participant will be set on an unweighting pressure gauge that controls a pneumatic cylinder attached to a compressor and a pulley a pulley system with minimal friction through an unloading harness to facilitate targeted 80% weight bearing throughout the entire gait cycle (h/p/cosmos airwalk®, h/p/cosmos sports & medical GmbH, Nussdorf-Traunstein, Germany). In the increased load condition, we will increase ambulatory load using an adjustable weight vest where approximately equal weight will be applied in the front and back. The added weight will total 20%BW for each participant to facilitate targeted 120% weight bearing throughout the entire gait cycle.

e) Ground reaction force

The walking stress test will be performed on an instrumented treadmill with an embedded plantar pressure plate (h/p/cosmos, Zebris FDM-T, Isny, Germany; 7168 sensors; area, 1.5 * 0.5 m; range, 1–120 N/cm^2; precision, 1-120 N/cm^2 ± 5%; sampling rate, 120 Hz). The vertical ground reaction impulse (area under the vertical GRF curve) times the number of steps taken during the walking stress test will be calculated. This measure will be used as surrogate measure for ambulatory load because tibiofemoral force is strongly correlated with walking speed and treadmill reaction forces.[34]

f) Joint kinematics and kinetics

For the walking stress test, an inertial sensor system (RehaGait®, Hasomed GmbH, Magdeburg, Germany) will be used to collect joint angle curves at the ankle, knee and hip. This system comprises seven inertial sensors[35] attached bilaterally on the lateral aspects of the shoes, the lower and upper leg, and on the pelvis. Each sensor contains a 3-axial accelerometer, gyroscope and magnetometer. Linear acceleration, angular velocity and the magnetic field are recorded at 400 Hz. All raw data can be exported, and the system calculates spatiotemporal gait parameters and joint kinematics (foot, ankle, knee, hip). In our previous studies, we found very good to good reliability[36,37] and good agreement with kinematic data obtained with the Vicon® system.[35] This data will be used to monitor joint kinematics for the three loading conditions to ensure that the general gait patterns remain the same among conditions and could be included in our statistical models if patterns change between conditions.

To compare the ankle, knee and hip kinematics and kinetics between subjects with ACL injury and subjects with healthy knees gait analysis will be carried out on the baseline day after the MRI was taken. The subjects will perform an instrumented gait analysis on a treadmill with an embedded plantar pressure plate (h/p/cosmos, Zebris FDM-T, Isny, Germany; 7168 sensors; area, 1.5 * 0.5 m; range, 1–120 N/cm^2; precision, 1-120 N/cm^2 ± 5%; sampling rate, 120 Hz) and on an overground walkway with two embedded force plates (Kistler force plate 9260AA6, Kistler AG, Winterthur, Switzerland; sampling rate 2400 Hz). Simultaneously with the plantar pressure or force data, kinematic and electromyographic (EMG) data will be collected using a 9 camera Vicon system (Vicon, Oxford, UK; frame rate 120 Hz) and a 12 channel EMG system (myon AG, Schwarzenberg, Switzerland, sampling rate 2400 Hz). To assess 3D joint angles, reflective markers will be placed on predefined anatomical landmarks on the pelvis and lower legs[38]. Surface electrodes will be placed bilaterally on the glutaeus medius, vastus medialis and lateralis, semitendinosus, tibialis anterior, and gastrocnemius medialis muscles following the guidelines of the SENIAM project (Surface ElectroMyoGraphy for the Non-Invasive Assessment of Muscles).[39] Participants will then walk barefoot for 2 minutes at 0% slope at their preferred walking speed and at 1 m/s. For all conditions, kinematic, EMG and pressure data will be recorded. Participants will put on their shoes. Subsequently, the treadmill speed will be increased to preferred running speed, and data for 2 minutes running will be recorded. Subjects will then complete overground walking trials in their own shoes on the walkway with embedded force plates. Maximum flexion, extension and rotation angles and joint moments will be computed using the Biomove software (Stanford University).[38,40] Intensity of the electromyographic signals of walking trials will be normalized to those for maximum voluntary contraction using a dynamometer (Biodex System 4 Pro: Biodex Medical Systems, Shirley, NY, USA).

g) Heart rate monitoring

To assess and compare the cardiovascular stress subjects experience during the walking stress tests the heart rate will be measured. Therefore, subjects will be asked to wear a heart rate monitor (Polar m400, Polar Electro Europe AG, Steinhausen, Switzerland) with a chest strap Polar H7, Polar Electro Europe AG, Steinhausen, Switzerland). From minute 40 to 42 in the first resting period the resting heart rate will be recorded. During the walking stress test until 10 minutes after the stress test, heart rate will be recorded. Mean heart rates will be used to compare the cardiovascular stress between the three loading conditions.

h) Biological assessment

Venous blood samples (5ml each) will be obtained from the antecubital vein at each evaluation. A vein catheter (Vasofix® Safety PUR 20G, B. Braun Melsungen AG, Melsungen Germany) will be placed during the rest period before the first blood sample at t_0 (Figure 1) and will stay there for the entire experiment (4.5 hours). After every blood sample, the catheter will be flushed with 10 ml isotonic saline solution (0.9% NaCl) to prevent plugging through clotting blood. The first 3 ml of every sample will be discarded to avoid dilution through the prior injected saline solution. Then the blood samples clot in the blood tubes (S-Monovette ® 7.5ml Z-Gel, Sarstedt AG, Nürnbrecht, Germany) for 30 minutes. Subsequently, they will be centrifuged (Sarstedt AG &Co SMC6) for 15 minutes at 2016 g and stored in the fridge (4°C) for no longer than 2 h until separation in aliquots at the Laboratory for Functional Biomechanics and frozen (-80°C). The concentration of serum biomarkers (IL-6, COMP, MMP-3, MMP-9, PGR-4, and ADAMTS-4) will be determined using commercially available ELISAs. Investigators will be blinded to the samples, which will be analyzed in duplicates and in random order. Differences due to inter-assay variation will be eliminated by comparing concentrations within subjects and testing all samples of any subject on the same plate. All blood analysis will be carried out by a professional service (TecoMedical). The service provider will not receive any information on the samples other than the subject code and random sample numbers to ensure that all samples of the same subject will be placed on the same plate. The change in blood markers of articular cartilage will be calculated as the serum concentration after the walking exercise normalized to the serum concentration before the walking exercise.

i) Tissue status

Advanced MRI techniques offer quantitative assessment of macromolecular changes to the cartilage matrix.[41,42] T2 relaxation time mapping has demonstrated some ability to track cartilage quality in OA[42], and T2 relaxation times are elevated in patients following ACL injury as early as 6 months after injury.[43] Moreover, we have previously used MRI data to study the effect of unloading during bed-rest on cartilage morphology of in vivo tibiofemoral cartilage.[44] Here, we will use T2 relaxation time as surrogate measure for assessing cartilage tissue status. MRI of both knees will be obtained using a 3T MR scanner (Prisma, Siemens Healthineers, Erlangen, Germany). For MRI measurements, subjects will be positioned supine and entered feet first. Each knee will be scanned separately. The knee will be centered in a dedicated knee coil, the popliteal fossa padded, allowing a slight knee flexion. The articular cartilage of the tibiofemoral joint will be imaged in the sagittal plane. Imaging will be performed with a custom 5 minute 3D DESS sequence[10] (echo time S+ (TE S+)= 5.7msec, echo time S- (TE S-)= 30.1msec, repetition time (TR)=17.9msec, flip angle (FA)=20°, field of view (FOV)= 170 mm^2, slice thickness (ST)= 1.5 mm, matrix (RO x PE) 384 x 512; pixel size (PS)= 0.42 x 0.31mm^2, approx. 5 min). MRI segmentation will be done by an independent company (Chondrometrics GmbH medical data processing, Ulrichshögelstrasse 23 D, 83404 Ainring, Germany) using their custom software (WORKS 3.0). The service provider will not receive any information on the images other than the subject code and random sample numbers to ensure that all samples of the same subject will be assessed in the same evaluation session. To measure differences in cartilage thickness in the tibiofemoral weight bearing regions, the tibiofemoral contact areas in the femoral cartilage will be divided into three functional weight bearing regions for each condyle based on the knee flexion angle during walking.[44,45] The average thickness will be calculated for the weight bearing regions on the femur and tibia. T2 relaxation time is used as surrogate measure to assess cartilage tissue status. Cartilage degeneration will be assessed as the difference in T2 relaxation times and cartilage thickness between baseline and 24-month follow-up, respectively. The articular cartilage of the knee joint will be defined as normal if the cartilage thickness is preserved, no cartilage signal alterations and no superficial and deep cartilage defects or fissures are present.

j) Other parameters

Information regarding treatment (conservative or operative), time since injury, time of operation (if applicable), duration of physiotherapy, time of return to work, time of return to sport, or type of sports before injury will be recorded from the patient file.

3.4 Withdrawal and discontinuation

Participants will be excluded if they withdraw their informed consent or do not follow the instructions given in the participant information. Data collected (clinical, biomechanical, biological) before withdrawal or discontinuation will be included in subsequent analyses to account for potential data bias. It is not possible to anonymise data in case a subject withdraws their consent to participate, and hence all data collected before withdrawal will be still be coded.

4 STATISTICS AND METHODOLOGY

4.1. Statistical analysis plan

All statistical analyses will be performed in Stata 15 (StataCorp LLC, College Station, Texas). In addressing the primary objective we will make use of the following variables:

- measurements of the blood markers at the five time points t_0, t_1, t_2, t_3 and t_4 under the three conditions;

- the actual ambulatory load at each condition as determined by the biomechanical assessment;
- age of the participant;
- tissue status at baseline: T2 relaxation time and cartilage thickness;
- inflammation status at baseline.

We will start with an initial data analysis to inform the following research question driven analyses without touching these questions. The following aspects will be addressed:
- The marginal distribution of all variables.
- The association between the factors age, T2 relaxation time, cartilage thickness and inflammation.
- The typical course over time for each biomarker in order to define two (or more) outcomes reflecting the initial change (t_1 compared to t_0) and the following movement back to the initial level (course from t_1 over t_2 to t_4).
- Variance heterogeneity and normality of residuals over time.
- Linearity of change in outcomes in dependence on ambulatory load.
- Variance heterogeneity of the outcomes in dependence on ambulatory load, age, tissue status and inflammation status.
- Correlations across biomarkers with respect to original measurements and outcomes.
- Characterization of the bivariate distribution of the two outcomes.

The research question will be addressed by the following sequence of analyses:
1. We will investigate the sensitivity of each outcome to the change in ambulatory load as well as the biological variation of the corresponding slope by a mixed model with the intercept and load as fixed and random factors. We will report:
   - the sensitivity of each biomarker to an ambulatory load, i.e. the population mean and population variation of the initial change at a load of 100%;
   - the sensitivity of each biomarker to a change in ambulatory load, i.e. the population mean of the slope of the regression of the initial change on the ambulatory load; and
   - the biological variation of these slopes.

   In addition, the results will be stratified by age, tissue status and inflammation.
2. We will investigate the effect of age on the slope for each outcome by a mixed model with intercept, load, age and the interaction between age and load as fixed effects and intercept and load as random effects.
3. We will investigate the effect of tissue and inflammation status (in addition to age) on the slope by a mixed model with intercept, load, age, tissue status, inflammation status and the interaction between the load and age, tissue status and inflammation status, respectively, as fixed effects and intercept and load as random effects. In dependence of the correlation between T2 relaxation time and cartilage thickness we will fit a separate model for both aspects of tissue status or a joint model.
4. We will investigate the correlation between the slopes for the two outcomes by corresponding joint mixed models and investigate also a dependence of the correlation on age, tissue status and inflammation status.
5. We will investigate the correlation of the slopes across the five different biomarkers by corresponding joint mixed models.

To allow simple comparisons of effects across the different biomarkers we will rescale all biomarkers to a median level of 100 at t_0 under 100% intended ambulatory load. The dependence of the slope on age, tissue status and inflammation status will be expressed as the expected slope at selected values, e.g. lower and upper 10% population percentiles. The value of these factors will be depicted by the explained biological variation. To take the joint investigation of five – however probably correlated – biomarkers into account, we will use a reduced significance level of 0.025. Further explanatory and hypotheses generating analyses are planned with respect to

new definitions of potential markers (e.g. ratios between two markers) or alternative definition of explaining factors (e.g. the three single inflammation markers).

With respect to the secondary objective, we will make use of additional clinical outcomes measured both a baseline and at the 2-years follow up. The primary outcome will be the change in T2 relaxation time. Secondary outcomes are the initial gait analysis, the change in cartilage thickness, KOOS score, modified KSS score and physical activity. The distribution of the outcomes will be depicted in an initial data analysis. The research questions will be addressed by the following sequence of analyses:

1. We will investigate for each biomarker the associations of the individual slope with the clinical outcomes by a corresponding regression model focusing on effect sizes and explained variation.
2. We will investigate the independent prognostic value of the individual slopes for each biomarker by corresponding adjustments, first for age and then also for tissue status and inflammation.
3. We will investigate a potential effect modification by physical activity by adding corresponding interactions terms to the regression models.
4. In case the individual slopes do not carry independent prognostic value, we will investigate their mediation effects on the path from age (or age, tissue status and inflammation) towards the clinical outcome.
5. In case of independent prognostic value of the individual slopes for several biomarkers, we will also consider the amount of independent prognostic information by considering a model with all these biomarkers as covariates.

In all regression models the baseline value of the clinical outcome will be included as covariate. Since the individual slopes are estimated and we can assess their standard error, we will correct for this measurement error using regression calibration.

Sample size calculation

To judge the adequacy of the intended samples sizes, we performed simulation studies using the estimated population means, the estimated populations standard deviations (recently published for COMP[18]), and the estimated residual variance as true parameter values and assuming a correlation of 0.8 between intercept and slope. We then varied the population standard deviation of the slope by factors of 2 and 3 because we expect in our study a substantially larger population variation due to wider age range and including both diseased and healthy subjects. We conducted four simulation studies corresponding to the intended main analyses. All simulation studies are based on the planned sample size of 96 subjects and include 2500 repetitions per simulation.
With respect to *the primary objective*, the first simulation study looked at the standard error of the estimates of the population standard deviation of the slope. We observed standard errors of 1.87, 1.27, and 1.24 for population standard deviations of the slope of 3.7, 7.4 and 11.1, respectively. This suggests that we can obtain a rather precise picture of the population variation.
In the second simulation, we looked at the power to demonstrate a significant interaction between load and a single normally distributed covariate that can explain some of the biological variation of the slope. Already for a population standard deviation of 3.7, we obtained a power of 71% if the covariate can explain 50% of the biological variation. For a population standard deviation of 7.4, we reached a power of 85% if the covariate can explain at least 25% of the variation. For a population standard deviation of 11.1, we obtained a power of 89% if the covariate can explain at least 20% of the variation.
In the third simulation we considered the case of three normally distributed covariates with a correlation of 0.3, equal interaction terms with the load, and explaining together a certain percentage of the biological variation. We assumed here a moderate correlation of 0.3 to take into account that tissue status and inflammation status are both probably related to age and to each other. Our interest was the power to demonstrate a significant interaction with the load for one a priori selected covariate if the covariates together can indeed explain a large amount of a substantial population variation. For a population standard deviation of 7.4, we reached a power of 70% if the covariates explain together at least 82% of the variation, and for a population

standard deviation of 11.1 we reach a power of 80% if they explain together 74% of the variation. This suggests that we will be able to demonstrate interactions between tissue status/inflammation status and ambulatory load on top of the interaction with age if the interactions are of similar magnitude as those for age, if all three factors explain a substantial amount of the biological variation, and if we have a substantial population variation of the slope.

With respect to the secondary objective, we can use standard formulas to determine the power to demonstrate a significant association in dependence on the true correlation between the individual slopes and a clinical outcome. They indicate that we have a power of 72% to find a significant association if the true correlation is 0.3, and a power of 95% if the true correlation is 0.4, taking into account a potential drop out rate of 10%. With respect to the ability of assessing an independent prognostic value, we performed a simulation study taking up the scenario of the third simulation study above, but now focusing on the situation where age, tissue status and inflammation can explain the variation in the individual slopes only to a smaller degree of 40%. If we further assume that these three variables together can explain the variation in the clinical outcome to the same degree as the individual slopes, we obtain a power of 80% to demonstrate an independent prognostic value if the individual slopes have a partial correlation of 0.6 with the outcome.

Subject characteristics

To describe the characteristics of the subjects in each of the 4 subgroups descriptive statistic will be used. Mean and standard deviation of age, sex, body height, body mass, BMI, treatment (conservative or operative), time of operation (if applicable), time since injury, duration of physiotherapy, time of return to work, time of return to sport, or type of sports before injury will be calculated to describe the participating subjects. To monitor subjects for potential gait abnormalities the parameters from the gait analysis at baseline day will be used. In subjects with previous ACL injury (Group 2 and 4) method of treatment and time since injury will be recorded.

Parameters describing the walking stress test are mean walking speed, cadence, GRF, heart rate and gait kinematics and will be compared between loading conditions.

4.2. Handling of missing data

All data will be checked for quality and completeness at the time of testing. Hence, we do not expect missing data due to technical difficulties. Blood samples will be taken at specific time points. Complicacy during blood sampling and delayed draw times will be registered in the study protocol. To minimize drop-out before completion of the 3 test days, we will inform the participants in detail about the duration and content of each session. To prevent logistical time problems, we will set all appointments prior to the clinical assessment. Incomplete data sets will be included in the statistical analysis. Missing values will be replaced by the mean. Recruitment will be continued until 24 participants per group completed the baseline assessment and all three walking stress test days to ensure complete data sets for addressing the primary objective.

To minimize the drop-out rate during the 24-month follow-up for answering the secondary objective, we will contact the participants at 12-months as intermediate clinical follow-up (KOOS only). We will account for drop-outs by performing intent to treat analyses.

5 REGULATORY ASPECTS AND SAFETY

5.1 Local regulations / Declaration of Helsinki

This research project will be conducted in accordance with the protocol, the Declaration of Helsinki[3], the principles of Good Clinical Practice, the Human Research Act[2] (HRA) and the

Human Research Ordinance[1] (HRO) as well as other locally relevant regulations. The Project Leader acknowledges his responsibilities as both the Project Leader and the Sponsor.

5.2 Notification of safety and protective measures (HRO Art. 20)

The project leader is promptly notified (within 24 hours) if immediate safety and protective measures have to be taken during the conduct of the research project. The Ethics Committee will be notified via BASEC of these measures and of the circumstances necessitating them within 7 days.

5.3 Serious events (HRO Art. 21)

If a serious event occurs, the research project will be interrupted and the Ethics Committee notified on the circumstances via BASEC within 7 days according to HRO Art. 21[1].

5.4 Amendments

Substantial changes to the project set-up, the protocol and relevant project documents will be submitted to the Ethics Committee for approval according to HRO Art. 18 before implementation. Exceptions are measures that have to be taken immediately in order to protect the participants.

5.5 End of project

Upon project termination, the Ethics Committee will be notified within 90 days.

5.6 Insurance

Not applicable. In the event of project-related damage or injuries, the liability of the University Hospital Basel provides compensation, except for claims that arise from misconduct or gross negligence.

6 FURTHER ASPECTS

6.1 Overall ethical considerations

The total time subjects invest in this study is about 22 hours. The majority of this time will be spent resting while sitting in a chair where subjects can read, study or do some office work. We will accommodate the participants weekly schedule and offer also offer testing sessions outside of regular working hours. By participating in this study, participants will receive feedback on their joint healthy and function that may be useful for their individual physical activity level. There are practically no risk factors in this study (see 6.2).

6.2 Risk-Benefit Assessment

This project is a risk category A project. Collecting health-related personal data in this study entail minimal risks and burdens for the patient. The physical stress during the walking stress test is comparable with walking with a backpack. The venipuncture will be carried out by a study nurse and is a standard clinical assessment with low infection risk.[46] Double sided tape will used to

---

[1] A serious event is defined as any adverse event where it cannot be excluded, that the event is attributable to the sampling of biological material or the collection of health-related personal data, and which:
a. requires inpatient treatment not envisaged in the protocol or extends a current hospital stay;
b. results in permanent or significant incapacity or disability; or
c. is life-threatening or results in death.

attach skin markers on specific anatomic landmarks. The tape may cause some skin irritation similar to a standard band-aid. This study provides no direct benefit to the patient. However, we will inform patients of their specific joint health and gait pattern measured in this study.

The risk of a MRI of the knee joint can be neglected. This standardized examination is performed in daily clinical practice. No contrast media will be given. Patients with pacemakers, neurostimulators or other conditional MR devices will be excluded. All female patients prior to menopause have to complete a pregnancy test before the MRI scan. If the test is positive, the patient will be excluded.

This study provides no direct benefit to the patient. However, subjects receive a summary of their joint health status and ambulatory gait function as appreciation.

7 QUALITY CONTROL AND DATA PROTECTION

7.1 Quality measures

All study personnel will be trained on all important project related aspects and be able to conduct quality control during and immediately after data collection. This is necessary to minimize missing data due to technical difficulties. Data quality will be checked weekly by the project leader. Internal audits will be carried out upon request by the host institution.

Blood samples will be analysed in duplicates and in random order. Differences due to inter-assay variation will be eliminated by comparing concentrations within subjects and testing all samples of any subject on the same plate. Intra-essay variability is estimated as coefficient of variation between the duplicates.

For quality assurance the Ethics Committee may visit the research sites. Direct access to the source data and all project related files and documents must be granted on such occasions.

7.2 Data recording and source data

All data will be entered into a master data file created in Redcap®. Electronic raw data will be stored on a backup drive that will be stored in a locked cabinet. All paper documentation (consent forms, questionnaires, CRFs) will be stored in a locked cabinet.

7.3 Confidentiality and coding

Project data will be handled with uttermost discretion and is only accessible to authorized personnel who require the data to fulfil their duties within the scope of the research project. On the CRFs and other project specific documents, participants will only be identified by a unique participant number.

Health-related personal data will be coded using a sequentially selected code from a predefined list of 6-digit codes containing letters and numbers. The code will only be broken if it is necessary to avert and immediate risk to the health of the person concerned or to guarantee the rights of the person. Participants will be asked if they are willing to be contacted in the future for subsequent follow-ups not currently planned in this study. The key to the code and participants' contact information will be kept in a locked cabinet and only accessible by the project leader.

Biological material in this project is coded by the unique participant code. Biological material will be appropriately stored in a restricted area only accessible to authorized personnel. When blood samples are analysed by an extern institution blood samples will be sent with an appropriate parcel service ore are given directly to the company. The location of every blood sample is noted in the study log.

7.4 Retention and destruction of study data and biological material

The data obtained in this study will be used to establish a database. The data will be stored indefinitely. As specified above, the data will be coded and the identification key will be locked and only accessible by the project leader and project manager. Health related data will be stored for 10 years after publication of the research project.

When subjects have signed the informed consent blood samples will be stored indefinitely in a biobank and may be used for other studies. The samples may be reanalysed for additional markers as these become available and be used in potential future long-term follow-up studies. Should the biobank be terminated, the samples will be disposed as biological waste. As specified above, the data and biological samples will be coded, and the identification key will be locked and only accessible by the project leader and project manager.

8 FUNDING / PUBLICATION / DECLARATION OF INTEREST

This study is mainly funded the Swiss National Science Foundation (#320030_184912). Some salaries of team members are covered by the Department of Orthopaedics and Traumatology of the University Hospital Basel. The SNSF is not involved in any aspect of the project.

Local progress reports will be used to disseminate information obtained in this study to healthcare professionals at our institution. The results of this study will be published at scientific conferences and in peer-reviewed international scientific journals. Any publications resulting from this project will only contain coded data, and establishing a direct link between health related data and the participants will be precluded.

Interested third parties may contact the project leader. Data will not be shared with third parties with lower data protection standards than CH or EU. Only coded data may be shared.

We declare no conflict of interest.

9 REFERENCES

1. Council TSF. Ordinance on Human Research with the Exception of Clinical Trials. 2018:24.
2. Council TSF. Ordinance on Clinical Trials in Human Research 2014:40.
3. WMA Declaration of Helsinki – Ethical Principles for Medical Research Involving Human Subjects 2018; https://www.wma.net/policies-post/wma-declaration-of-helsinki-ethical-principles-for-medical-research-involving-human-subjects/. Accessed 02.07.19, 2019.
4. Woolf AD, Pfleger B. Burden of major musculoskeletal conditions. *Bull World Health Organ.* 2003;81(9):646-656.
5. E Ehrlich G. *The rise of osteoarthritis. Bull WHO 81: 630.* Vol 812003.
6. Kellgren JH, Lawrence JS. Radiological Assessment of Osteo-Arthrosis. *Annals of the Rheumatic Diseases.* 1957;16(4):494-502.
7. Disler DG. Invited Commentary. *RadioGraphics.* 2011;31(1):61-62.
8. Guermazi A, Roemer FW, Burstein D, Hayashi D. Why radiography should no longer be considered a surrogate outcome measure for longitudinal assessment of cartilage in knee osteoarthritis. *Arthritis Research & Therapy.* 2011;13(6):247.
9. Staroswiecki E, Granlund KL, Alley MT, Gold GE, Hargreaves BA. Simultaneous estimation of T2 and apparent diffusion coefficient in human articular cartilage in vivo with a modified three-dimensional double echo steady state (DESS) sequence at 3 T. *Magnetic Resonance in Medicine.* 2012;67(4):1086-1096.
10. Chaudhari AS, Black MS, Eijgenraam S, et al. Five-minute knee MRI for simultaneous morphometry and T2 relaxometry of cartilage and meniscus and for semiquantitative radiological assessment using double-echo in steady-state at 3T. *Journal of Magnetic Resonance Imaging.* 2018;47(5):1328-1341.

11. Mow VC, Proctor CS, Kelly MA. Biomechanics of Articular Cartilage. In: Nordin M, Frankel V, eds. *Basic Biomechanics of the Muskuloskeletal system*. 2nd ed. ed. Philadelphia: Lea and Febiger;; 1989.
12. Sophia Fox AJ, Bedi A, Rodeo SA. The basic science of articular cartilage: structure, composition, and function. *Sports Health.* 2009;1(6):461-468.
13. Dewan AK, Gibson MA, Elisseeff JH, Trice ME. Evolution of autologous chondrocyte repair and comparison to other cartilage repair techniques. *Biomed Res Int.* 2014;2014:272481.
14. Woo SLY, Buckwalter JA. Injury and repair of the musculoskeletal soft tissues. Savannah, Georgia, June 18–20, 1987. *J Orthop Res.* 1988;6(6):907-931.
15. Goldring MB. Osteoarthritis and cartilage: the role of cytokines. *Current Rheumatology Reports.* 2000;2(6):459-465.
16. Loeser RF, Goldring SR, Scanzello CR, Goldring MB. Osteoarthritis: a disease of the joint as an organ. *Arthritis and rheumatism.* 2012;64(6):1697-1707.
17. Sanchez-Adams J, Leddy HA, McNulty AL, O'Conor CJ, Guilak F. The mechanobiology of articular cartilage: bearing the burden of osteoarthritis. *Curr Rheumatol Rep.* 2014;16(10):451.
18. Herger S, Vach W, Liphardt AM, Egloff C, Nüesch C, Mündermann A. Dose-response relationship between ambulatory load magnitude and load-induced changes in COMP in young healthy adults. *Osteoarthritis and Cartilage.* 2019;27(1):106-113.
19. Mündermann A, Herger S, Vach W, Liphardt AM, Egloff C, Nüesch C. Dose-response relationship between ambulatory load magnitude and load-induced changes in serum biomarkers of cartilage health in young healthy adults. *Proceedings of the 11th Annual Meeting of the German Society of Biomechanics, April 2019, Berlin, Germany.* 2019.
20. Mündermann A, Klenk C, Billich C, et al. Changes in Cartilage Biomarker Levels During a Transcontinental Multistage Footrace Over 4486 km. *The American journal of sports medicine.* 2017;45(11):2630-2636.
21. Glasson SS, Askew R, Sheppard B, et al. Deletion of active ADAMTS5 prevents cartilage degradation in a murine model of osteoarthritis. *Nature.* 2005;434(7033):644-648.
22. Li W, Du C, Wang H, Zhang C. Increased serum ADAMTS-4 in knee osteoarthritis: a potential indicator for the diagnosis of osteoarthritis in early stages. *Genetics and molecular research : GMR.* 2014;13(4):9642-9649.
23. Schatti OR, Markova M, Torzilli PA, Gallo LM. Mechanical Loading of Cartilage Explants with Compression and Sliding Motion Modulates Gene Expression of Lubricin and Catabolic Enzymes. *Cartilage.* 2015;6(3):185-193.
24. Nelson F, Dahlberg L, Laverty S, et al. Evidence for altered synthesis of type II collagen in patients with osteoarthritis. *The Journal of Clinical Investigation.* 1998;102(12):2115-2125.
25. Poole AR, Ionescu M, Fitzcharles MA, Billinghurst RC. The assessment of cartilage degradation in vivo: development of an immunoassay for the measurement in body fluids of type II collagen cleaved by collagenases. *Journal of immunological methods.* 2004;294(1-2):145-153.
26. Szychlinska MA, Leonardi R, Al-Qahtani M, Mobasheri A, Musumeci G. Altered joint tribology in osteoarthritis: Reduced lubricin synthesis due to the inflammatory process. New horizons for therapeutic approaches. *Ann Phys Rehabil Med.* 2016;59(3):149-156.
27. Cooper C, Snow S, McAlindon TE, et al. Risk factors for the incidence and progression of radiographic knee osteoarthritis. *Arthritis and rheumatism.* 2000;43(5):995-1000.
28. Ackerman IN, Cavka B, Lippa J, Bucknill A. The feasibility of implementing the ICHOM Standard Set for Hip and Knee Osteoarthritis: a mixed-methods evaluation in public and private hospital settings. *J Patient Rep Outcomes.* 2017;2:32.
29. Insall JN, Dorr LD, Scott RD, Scott WN. Rationale of the Knee Society clinical rating system. *Clinical orthopaedics and related research.* 1989(248):13-14.
30. Roos EM, Lohmander LS. The Knee injury and Osteoarthritis Outcome Score (KOOS): from joint injury to osteoarthritis. *Health and Quality of Life Outcomes.* 2003;1(1):64.

31. Aadland E, Ylvisaker E. Reliability of the Actigraph GT3X+ Accelerometer in Adults under Free-Living Conditions. *PloS one.* 2015;10(8):e0134606.
32. Montoye AH, Pfeiffer KA, Suton D, Trost SG. Evaluating the Responsiveness of Accelerometry to Detect Change in Physical Activity. *Measurement in physical education and exercise science.* 2014;18(4):273-285.
33. Ozemek C, Kirschner MM, Wilkerson BS, Byun W, Kaminsky LA. Intermonitor reliability of the GT3X+ accelerometer at hip, wrist and ankle sites during activities of daily living. *Physiological measurement.* 2014;35(2):129-138.
34. Patil S, Steklov N, Bugbee WD, Goldberg T, Colwell CW, Jr., D'Lima DD. Anti-gravity treadmills are effective in reducing knee forces. *Journal of orthopaedic research : official publication of the Orthopaedic Research Society.* 2013;31(5):672-679.
35. Nüesch C, Roos E, Pagenstert G, Mündermann A. Measuring joint kinematics of treadmill walking and running: Comparison between an inertial sensor based system and a camera-based system. *J Biomech.* 2017;57:32-38.
36. Donath L, Faude O, Lichtenstein E, Nüesch C, Mündermann A. Validity and reliability of a portable gait analysis system for measuring spatiotemporal gait characteristics: comparison to an instrumented treadmill. *Journal of neuroengineering and rehabilitation.* 2016;13(1):6.
37. Donath L, Faude O, Lichtenstein E, Pagenstert G, Nüesch C, Mündermann A. Mobile inertial sensor based gait analysis: Validity and reliability of spatiotemporal gait characteristics in healthy seniors. *Gait & posture.* 2016;49:371-374.
38. Dyrby CO, Andriacchi TP. Secondary motions of the knee during weight bearing and non-weight bearing activities. *J Orthop Res.* 2004;22(4):794-800.
39. Hermens HJ, Freriks B, Disselhorst-Klug C, Rau G. Development of recommendations for SEMG sensors and sensor placement procedures. *J Electromyogr Kinesiol.* 2000;10(5):361-374.
40. Grood ES, Suntay WJ. A joint coordinate system for the clinical description of three-dimensional motions: application to the knee. *J Biomech Eng.* 1983;105(2):136-144.
41. Regatte RR, Akella SV, Borthakur A, Kneeland JB, Reddy R. Proteoglycan depletion-induced changes in transverse relaxation maps of cartilage: comparison of T2 and T1rho. *Acad Radiol.* 2002;9(12):1388-1394.
42. Stahl R, Blumenkrantz G, Carballido-Gamio J, et al. MRI-derived T2 relaxation times and cartilage morphometry of the tibio-femoral joint in subjects with and without osteoarthritis during a 1-year follow-up. *Osteoarthritis and cartilage / OARS, Osteoarthritis Research Society.* 2007;15(11):1225-1234.
43. Monu UD, Jordan CD, Samuelson BL, Hargreaves BA, Gold GE, McWalter EJ. Cluster analysis of quantitative MRI T2 and T1rho relaxation times of cartilage identifies differences between healthy and ACL-injured individuals at 3T. *Osteoarthritis and cartilage / OARS, Osteoarthritis Research Society.* 2016.
44. Liphardt AM, Mündermann A, Koo S, et al. Vibration training intervention to maintain cartilage thickness and serum concentrations of cartilage oligometric matrix protein (COMP) during immobilization. *Osteoarthritis and Cartilage.* 2009;17(12):1598-1603.
45. Koo S, Gold GE, Andriacchi TP. Considerations in measuring cartilage thickness using MRI: factors influencing reproducibility and accuracy. *Osteoarthritis and Cartilage.* 2005;13(9):782-789.
46. Shah H, Bosch W, Thompson KM, Hellinger WC. Intravascular Catheter-Related Bloodstream Infection. *The Neurohospitalist.* 2013;3(3):144-151.
