# Supplementary material for: Dose-response relationship of in vivo ambulatory load and mechanosensitive cartilage biomarkers—The role of age, tissue health and inflammation: A study protocol
Source: PLoS One. 2022 Aug 19;17(8):e0272694. doi: 10.1371/journal.pone.0272694 (PMC9390933; doi:10.1371/journal.pone.0272694)
Supplement: S3 File — (PDF) [file pone.0272694.s003.pdf]

[redacted]

[redacted]

Präsident
Prof. Christoph Beglinger
Vizepräsidenten
Dr. Angela Frotzler
Dr. Marco Schärer

Prof. Dr. Annegret Mündermann
University Hospital Basel
Orthopaedics and Traumatology
Spitalstrasse 21
4031 Basel

Basel, 16. August 2019

Verfügung der Ethikkommission Nordwest- und Zentralschweiz (EKNZ)

**Project-ID**	2019-01315
**Projekttitel**	MechSens - Dose-response relationship of in vivo ambulatory load and mechanosensitive cartilage biomarkers: the role of age and tissue health
**Master-/Doktorarbeit von**	Herger, Simon
**Haupt-Prüfer / Koordinierender Prüfer**	Prof. Dr. Annegret Mündermann
**Sponsor**	Prof. Dr. Annegret Mündermann
**Zentren**	• Prof. Dr. Annegret Mündermann, University Hospital Basel, Basel

Entscheid

☒ Die Bewilligung wird erteilt → die Auflagen der EKNZ vom 12. August 2019 wurden erfüllt.
☐ Die Bewilligung wird mit Auflagen erteilt
☐ Die Bewilligung kann noch nicht erteilt werden
☐ Die Bewilligung wird nicht erteilt
☐ Auf das Gesuch wird nicht eingetreten

Klassifizierung

☒ Forschungsprojekt gemäss HFV — Kategorie: A
- ☒ Forschung mit Personen
- ☐ Weiterverwendung des biologischen Materials oder der gesundheitsbezogenen Personendaten
- ☐ mit Verstorbenen
- ☐ mit Embryonen / Föten
- ☐ mit ionisierender Strahlung

Entscheidverfahren

☐ ordentliches Verfahren ☐ vereinfachtes Verfahren ☒ Präsidialverfahren

Die Ethikkommission bestätigt, dass sie nach ICH-GCP arbeitet.

Gebühren

Betrag: **CHF** **Tarifcode:**

Gemäss der geltenden Gebührenordnung von swissethics.

Rechtsmittelbelehrung

Gegen diesen Entscheid kann an den Regierungsrat des Kantons Basel-Stadt (Rathaus, Marktplatz 9, 4051 Basel) rekurriert werden. Der Rekurs ist innert 10 Tagen seit Eröffnung des Entscheides bei der Rekursinstanz anzumelden; innert 30 Tagen, vom gleichen Zeitpunkt an gerechnet, ist die Rekursbegründung einzureichen, welche die Anträge und deren Begründung mit Angabe der Beweismittel zu enthalten hat. Bei völliger oder teilweiser Abweisung des Rekurses können die Kosten der Rekurrentin respektive dem Rekurrenten ganz oder teilweise auferlegt werden

Kopie an

☐ BAG

☒ Andere

Simon Herger, simon.herger@usb.ch
Corina Nüesch, Corina.nueesch@usb.ch
Ilona Ahlborn, ilona.ahlborn@usb.ch

Unterschrift

[redacted]

Prof. Dr. med. Christoph Beglinger
Präsident

Anhang:
1. Pflichten des Sponsors/der Prüfperson oder der Projektleitung
2. Mögliche Entscheide und ihre Bedeutung
3. Eingereichte Dokumente (15.08.2019)

Anhang 1

Pflichten des Sponsors/der Prüfperson oder der Projektleitung:

Einreichung Dokumente: revidierte Dokumente und neue Dokumente zur Studie/zum Projekt sollen ausschliesslich über das Web-Portal BASEC eingereicht werden, auf der entsprechenden Formularseite des betreffenden Gesuches. Obsolete Dokumente sind dabei zu entfernen und Datums- und Versionsangaben entsprechend zu ergänzen. Die erfolgten Änderungen müssen im Korrekturmodus abgefasst werden und zusätzlich als ‚clean'-Version eingereicht werden. Die Studieninformationen und -einwilligungen, das Protokoll und die Amendments müssen in durchsuchbaren PDF-Dateien eingereicht werden, insbesondere müssen gescannte Dokumente eine Texterkennung durchlaufen haben (OCR). Das unterschriebene und datierte Begleitschreiben muss die Antworten auf eventuell von der EK gestellte Fragen enthalten. Revidierte Dokumente sind auch den weiteren Zulassungsbehörden zuzustellen, sofern diese involviert sind.

<u>Anmerkung</u>: Die zuständige Ethikkommission überprüft im Rahmen des Bewilligungsverfahrens Aufklärungsbo-

gen und Einwilligungserklärung in einer der Amtssprachen Deutsch, Französisch oder Italienisch. Aufklärungsbogen und Einwilligungserklärung in einer anderen Sprache werden von der Ethikkommission lediglich zur Kenntnis genommen. Für die korrekte Übersetzung ist der Sponsor oder die Projektleitung verantwortlich.

Meldepflichten: Die rechtlich bindenden Melde- resp. Bewilligungspflichten an die Ethikkommission für wesentliche Änderungen, einen vorzeitigen Studienabbruch, unerwünschte Ereignisse u.a. sind einzuhalten (Verordnungen des Bundes). Der Abschlussbericht ist spätesten ein Jahr nach Studienende der Ethikkommission einzureichen.

Registrierungspflicht: Der Sponsor muss – falls es sich um einen klinischen Versuch handelt – diesen in einem WHO-Primärregister oder im Register der Nationalen Medizinbibliothek der USA (clinicaltrials.gov) erfassen und anschliessend diese Nummer im BASEC-Portal eingeben. Die Übertragung der erforderlichen Daten in das Swiss National Clinical Trials Portal (SNCTP) kann nach Bewilligung der Ethikkommission und Zustimmung des Gesuchstellers automatisch erfolgen. Die Informationen über den klinischen Versuch sind in beiden Registern öffentlich zugänglich. Zusätzlich veröffentlicht swissethics wenige Informationen wie Titel, Projekttyp oder Leit-Ethikkommission aller durch die kantonalen Ethikkommissionen bewilligten Gesuche auf swissethics.ch (ausser Phase-I-Studien).

Anhang 2

Mögliche Entscheide und ihre Bedeutung

Die Bewilligung wird erteilt: Das Vorhaben kann gemäss bewilligtem Forschungsplan und im Rahmen der anwendbaren rechtlichen Bestimmungen durchgeführt werden. Weitere Bewilligungspflichten (Swissmedic/BAG) sind zu beachten

Die Bewilligung wird mit Auflagen erteilt: Das Vorhaben kann gemäss bewilligtem Forschungsplan gestartet werden und im Rahmen der anwendbaren rechtlichen Bestimmungen durchgeführt werden. Die Auflagen sind zu erfüllen und die Gesuchsunterlagen innert 30 Tagen entsprechend anzupassen. Die revidierten Dokumente werden nach Einreichung im Präsidialverfahren geprüft. Weitere Bewilligungspflichten (Swissmedic/BAG) sind zu beachten

Die Bewilligung kann noch nicht erteilt werden: Das Vorhaben kann noch nicht gestartet werden. Die nachfolgenden Bedingungen sind zu erfüllen bzw. die Fragen zu beantworten und die revidierten Dokumente erneut bei der Ethikkommission einzureichen. Die Ethikkommission überprüft die revidierten Dokumente und erteilt die Bewilligung, wenn die Bedingungen erfüllt bzw. die Fragen zufriedenstellend beantwortet sind.

Die Bewilligung wird nicht erteilt: Das Vorhaben kann in der vorliegenden Form nicht durchgeführt werden. Eine Neueinreichung ist möglich.

Auf das Gesuch wird nicht eingetreten: Begründung siehe vorne, z.B. nicht zuständig oder nicht bewilligungspflichtig.

Anhang 3

Eingereichte Dokumente für das Hauptzentrum

Prof. Dr. Annegret Mündermann, University Hospital Basel, Basel

Dokument	Dok.Datum	Version
mechsens-begleitschreiben-v2-2019-08-13.pdf	15.07.2019	
mechsens-pic-gesund-v1-2019-08-13-tracked.docx	13.08.2019	1
mechsens-pic-gesund-v1-2019-08-13.pdf	13.08.2019	1
mechsens-pic-v2-2019-08-13-tracked.docx	13.08.2019	2
mechsens-pic-v2-2019-08-13.pdf	13.08.2019	2
mechsens-studprot-v2-2019-08-13-tracked.docx	13.08.2019	2
mechsens-studprot-v2-2019-08-13-signed.pdf	13.08.2019	2
mechsens-studprot-v2-2019-08-13.pdf	13.08.2019	2
mechsens-flyer-v2-2019-08-13-tracked.docx	13.08.2019	2
mechsens-flyer-v2-2019-08-13.pdf	13.08.2019	2
