# Supplementary material for: Dose-response relationship of in vivo ambulatory load and mechanosensitive cartilage biomarkers—The role of age, tissue health and inflammation: A study protocol
Source: PLoS One. 2022 Aug 19;17(8):e0272694. doi: 10.1371/journal.pone.0272694 (PMC9390933; doi:10.1371/journal.pone.0272694)
Supplement: S5 File — (PDF) [file pone.0272694.s005.pdf]

[redacted]

[redacted]

Präsident
Prof. Christoph Beglinger
Vizepräsidenten
Dr. Angela Frotzler
Dr. Marco Schärer

Prof. Dr. Annegret Mündermann
University Hospital Basel
Orthopaedics and Traumatology
Spitalstrasse 21
4031 Basel

Basel, 04. Januar 2022

## Verfügung der Ethikkommission Nordwest- und Zentralschweiz (EKNZ)

| | |
|---|---|
| **Wesentliche Änderung** | **Amendment 02 vom 09. Dezember 2021 (Protokoll Version 5)** |
| **eingereicht am** | 03.01.2022 |
| **Project-ID** | 2019-01315 |
| **Projekttitel** | MechSens - Dose-response relationship of in vivo ambulatory load and mechanosensitive cartilage biomarkers: the role of age and tissue health |
| **Master-/Doktorarbeit von** | Herger, Simon |
| **Projektleitung** | Prof. Dr. Annegret Mündermann |
| **Sponsor** | Prof. Dr. Annegret Mündermann |
| **Zentren** | Prof. Dr. Annegret Mündermann, University Hospital Basel, Basel |

### Entscheidverfahren

☐ vereinfachtes Verfahren ☒ Präsidialverfahren

### Entscheid

**Prof. Dr. Annegret Mündermann, University Hospital Basel, Basel**

☒ Die Bewilligung wird erteilt
☐ Die Bewilligung wird mit Auflagen erteilt
☐ Die Bewilligung kann noch nicht erteilt werden
☐ Die Bewilligung wird nicht erteilt

### Gebühren

**Betrag:** **CHF 250.--** **Tarifcode:** **3.3.1**
Gemäss der geltenden Gebührenordnung von swissethics.

### Rechtsmittelbelehrung

Gegen diesen Entscheid kann an den Regierungsrat des Kantons Basel-Stadt (Rathaus, Marktplatz 9, 4051 Basel) rekurriert werden. Der Rekurs ist innert 10 Tagen seit Eröffnung des Entscheides bei der Rekursinstanz anzumelden; innert 30 Tagen, vom gleichen Zeitpunkt an gerechnet, ist die Rekursbegründung einzureichen, welche die Anträge und deren Begründung mit Angabe der Beweismittel zu enthalten hat. Bei völliger oder teilweiser Abweisung des Rekurses können die Kosten der Rekurrentin respektive dem Rekurrenten ganz oder teilweise auferlegt werden.

## Kopie an

☐ BAG
☒ Andere — Simon Herger, simon.herger@usb.ch
Corina Nüesch, Corina.nueesch@usb.ch
Ilona Ahlborn, ilona.ahlborn@usb.ch

Die Ethikkommission bestätigt, dass sie nach ICH-GCP arbeitet.

## Unterschriften

[redacted]

Prof. Dr. med. Christoph Beglinger
Präsident

**Anhang:**
1. Bedeutung der möglichen Entscheide
2. Liste der Dokumente, eingereicht am 03.01.2022

## Anhang 1

### Bedeutung der möglichen Entscheide

**Die Bewilligung wird erteilt**: Das Vorhaben kann gemäss der oben aufgeführten wesentlichen Änderung weitergeführt werden.

## Anhang 2

### Liste der Dokumente, eingereicht am 03.01.2022

**Prof. Dr. Annegret Mündermann, University Hospital Basel, Basel**

| Dokument | Kategorie | Dok.Datum | Version |
|---|---|---|---|
| mechsens-begleitschreiben-v6-2021-12-12.pdf | 1. Cover Letter | 12/12/2021 | |
| mechsens-pic-gesund-v4-1-2021-12-09-clean.pdf | 3. ICF | 09/12/2021 | 4.1 |
| mechsens-pic-vkb-v4-2-2021-12-09-clean.pdf | 3. ICF | 09/12/2021 | 4.2 |
| mechsens-pic-vkb-v4-2-2021-12-09-tracked.docx | 3. ICF | 09/12/2021 | 4.2 |
| mechsens-pic-gesund-v4-1-2021-12-09-tracked.docx | 3. ICF | 09/12/2021 | 4.1 |
| mechsens-studprot-v5-2021-12-09-tracked.docx | 4. Study plan | 09/12/2021 | 5 |
| mechsens-studprot-v5-2021-12-09-signed.pdf | 4. Study plan | 09/12/2021 | 5 |
| mechsens-studprot-v4-1-2020-01-17-tracked.docx | 4. Study plan | 17/01/2020 | 4.1 |
