# Supplementary material for: Dose-response relationship of in vivo ambulatory load and mechanosensitive cartilage biomarkers—The role of age, tissue health and inflammation: A study protocol
Source: PLoS One. 2022 Aug 19;17(8):e0272694. doi: 10.1371/journal.pone.0272694 (PMC9390933; doi:10.1371/journal.pone.0272694)
Supplement: S6 File — (PDF) [file pone.0272694.s006.pdf]

Swiss National
Science Foundation

Unit Projects Life Sciences
Wildhainweg 3, P.O. Box
3001 Berne

+41 31 308 22 22
Projects.ls@snf.ch
www.snsf.ch

To whom it may concern

Berne, 17 June 2022

320030_184912

The Swiss National Science Foundation SNSF is the main public funding agency for academic research in Switzerland.

We herewith confirm that the study protocol for the project 32003B_184912 "Dose-response relationship of in vivo ambulatory load and cartilage biomarkers: the role of age, tissue health and inflammation" was selected for funding after independent peer review. The SNSF has no self-interest in the study outcomes.

Yours sincerely

Christoph Meier, PhD
Head of Unit Projects Life Sciences
